# Improving student diet and food security in higher education using participatory and co-creation approaches: a systematic review

**DOI:** 10.1186/s12966-024-01613-7

**Published:** 2024-07-08

**Authors:** Tamar Assilian, Henri Dehove, Hélène Charreire, Julia Baudry, Emmanuelle Kesse-Guyot, Sandrine Péneau, Chantal Julia, Olivia Gross, Jean-Michel Oppert, Alice Bellicha

**Affiliations:** 1 Center of Research in Epidemiology and StatisticS (CRESS), Nutritional Epidemiology Research Team (EREN), Université Sorbonne Paris Nord and Université Paris Cité, INSERM, INRAE, CNAM, Bobigny, 93017 France; 2 MoISA, Univ Montpellier, CIRAD, CIHEAM-IAMM, INRAE, Institut Agro, IRD, Montpellier, France; 3 Univ Paris Est Créteil, LabUrba, Créteil, 94010 France; 4 Health Education and Practices Laboratory, University Sorbonne Paris Nord, Bobigny, 93017 France; 5 Department of Nutrition, Pitié-Salpêtrière Hospital, Assistance Publique-Hôpitaux de Paris (AP-HP), Sorbonne University, CRNH-Ile de France, Paris, France

**Keywords:** Young adults, Students in higher education, Intervention, Participatory approach, Co-creation, Systematic review

## Abstract

**Background:**

Higher education students are an important target group for public health nutrition interventions. When designing tailored and contextually relevant interventions, participatory and co-creation approaches are increasingly recognized as promising but their use and effectiveness has not been assessed in this type of population. We systematically reviewed interventions aiming to improve dietary quality and/or food security in higher education settings with the aims 1) to identify and describe their participatory and co-creation approaches and 2) to compare the effectiveness of interventions using or not using participatory and co-creation approaches.

**Methods:**

Our search in PubMed, Google Scholar, Web of Science, EMBASE was performed in January 2023 and yielded 3658 unique records, out of which 42 articles (66 interventions) were included. Effectiveness of interventions was assessed at the individual level (longitudinal evaluations) or at the group level (repeated cross-sectional evaluations). A five-level classification was used to describe a continuum of engagement from students and other partners in the intervention design and implementation: no participation (level one), consultation, co-production, co-design and co-creation (levels two to five). To synthetize effectiveness, comparisons were made between studies without participation (level one) or with participation (levels two-five).

**Results:**

Ten (24%) out of 42 studies used a participatory and co-creation approach (levels two-five). Studies using a participatory and co-creation approach reported a positive finding on individual-level outcome (i.e. overall diet quality or food group intake or food security) in 5/13 (38%) intervention arms (*vs* 13/31 or 42% for those without participation). Studies using a participatory and co-creation approach reported a positive finding on group-level outcomes (i.e. food choices in campus food outlets) in 4/7 (57%) (*vs* 8/23 or 35% in those without participation).

**Conclusions:**

Participatory and co-creation approaches may improve the effectiveness of nutrition interventions in higher education settings but the level of evidence remains very limited. More research is warranted to identify best co-creation practices when designing, implementing and evaluating nutritional interventions in the higher education setting.

**Trial registration:**

PROSPERO registration number CRD42023393004.

**Supplementary Information:**

The online version contains supplementary material available at 10.1186/s12966-024-01613-7.

## Background

Unhealthy diets are among the leading contributors to non-communicable diseases morbidity and mortality worldwide [1]. Young adults, i.e. those aged 18–25 y, including the student population enrolled in higher education, are considered an important target group for public health nutrition interventions, in particular because the transition period from adolescence to young adulthood is critical for developing new health behaviors [2, 3]. However, this period of life appears increasingly characterized by a shift towards less healthy dietary intakes [2, 3], a disruption of eating behavior patterns [4] and weight gain [5]. Rates of food insecurity, defined as a lack of access to adequate food to support a healthy and active lifestyle [6], are also high in higher education students, with potential detrimental consequences on academic achievement as well as physical and emotional health [7–9]. In a recent scoping review, 41% of higher education students in the US reported food insecurity, a proportion higher than the national average of 10% [10, 11]. Identifying interventions likely to improve diet quality and food security of students is therefore a relevant and timely objective.

Barriers to healthy eating habits and food security in higher education students encompass lack of time, poor cooking skills and limited financial resources [12–14], but also unhealthy campus food environments [15–17]. Broadly defined as “the collective physical, economic, policy and sociocultural surroundings, opportunities and conditions that influence people’s food and beverage choices and nutritional status” [18], the food environment is recognized as a major determinant of diet. Prior reviews have synthetized literature on interventions intended to promote healthy eating habits or food security in this setting [8, 19–22]. Overall results of these reviews provided no clear evidence that on-campus interventions improve students’ diet [8, 19–22]. Education interventions were found to be effective to improve nutritional quality of dietary intakes in less than 50% of studies [19]. Reviews of interventions targeting the campus food environment showed large variability in results, with positive findings on improved diet quality or food purchases reported in 47% [19], 58% [22] or 87% [20] of studies. Interventions addressing food insecurity using multiple strategies such as providing nutrition education, recipes, meal and produce vouchers, or access to food charity may improve the diet quality of food-insecure students [8].

Importantly, these reviews have not considered how and by whom the intervention was designed and implemented. Involving those who are intended to be the beneficiaries and partners of public health interventions is however considered critical to design tailored, pragmatic and contextually relevant interventions [23, 24]. Co-creation promotes the engagement of beneficiaries and partners in the design, implementation and evaluation of interventions, and thus aligns with the fundamental principle of participatory research [25]. In participatory research, the persons whose life or work is the subject of the research (e.g. citizens, patients, community members, professionals or institutional representatives) partner with academics and actively take part to the research process [23]. This partnership can take many forms and can lead to shared decision-making between academic and non-academic actors in the deepest forms of participation [25, 26]. Participatory research is increasingly recognized as a promising approach to improve the relevance and suitability of research questions, to better meet the needs and expectations of target populations, and to favor community engagement which, subsequently, could improve the effectiveness of interventions [27–29]. In the field of public health nutrition, the number of intervention studies that have used participatory and co-creation approaches to improve the quality of dietary intakes or the food environment remains limited, with however some evidence of the benefits of co-creation [30, 31]. To date, the application of participatory research and co-creation in higher education settings, and its potential added value in improving students’ diet, has not been systematically synthesized.

To address this knowledge gap, we conducted a systematic review with the aims 1) to identify and describe participatory and co-creation approaches used in interventions which aim to improve dietary quality and/or food security in higher education settings and 2) to evaluate their effectiveness compared to those without participation and co-creation approach.

## Methods

This systematic review follows the Preferred Reporting Items for Systematic Reviews and Meta-Analysis (PRISMA) guidelines and is registered in PROSPERO (CRD42023393004).

### Literature search and selection of studies

We systematically searched PubMed, Google Scholar, Web of Science and EMBASE in January 2023 using a combination of terms related to students, diet, intervention and participation (Supplementary Table 1). We also manually scanned the reference sections of the included original papers and reviews for further eligible studies.

We included papers written in English and published in peer-reviewed journals since January 2013 if they met the following inclusion criteria: 1) an intervention aiming at improving dietary intakes, food purchases and/or food security was conducted in a higher education (i.e. post-secondary education) setting, 2) the intervention targeted students dietary behaviors and/or the campus food environment, 3) the study design was controlled when the intervention targeted individual behaviors, and controlled or based on an interrupted time-series design when the intervention targeted the campus food environment, and 4) outcomes were related to diet quality (overall diet quality score or consumption of food groups considered as healthy or unhealthy), food security, or food choices in campus food outlets (campus restaurant, cafeteria, vending machines). We included studies using, or not, participatory and co-creation approaches to be able to compare the effectiveness of studies with and without participation and-co-creation.

Combined interventions aiming at improving dietary intakes and other health behaviors (e.g., physical activity, stress, smoking…) were included. Studies targeting a specific group of students (e.g., with eating disorders, type 2 diabetes…), or assessing only eating disorders, intentions to change dietary intakes, or sustainable eating practices such as food waste were not included. Finally, studies targeting both students and university staff without stratifying on population type in analyses were not included. Abstracts and full texts were assessed for eligibility by two authors independently (TA and AB). Any disagreement between reviewers was resolved through discussion.

### Data extraction

Data were extracted by one author (TA) using standardized forms and then checked by another author (AB). The characteristics of each article included: country, study design, population characteristics, description of intervention topic and strategy, outcome variables, assessment methods and findings. Emphasis was placed on the description of interventions. The Template for Intervention Description and Replication (TIDierR) checklist [32] (including the following items: why, what materials and procedures, who provided, how, where, when and how much, tailoring, modification of intervention throughout trial, strategies to improve or maintain intervention fidelity and extent of intervention fidelity) was used for description of intervention and control conditions (Supplementary Table 2).

### Classification of participatory and co-creation approaches

**Fig. 1:**
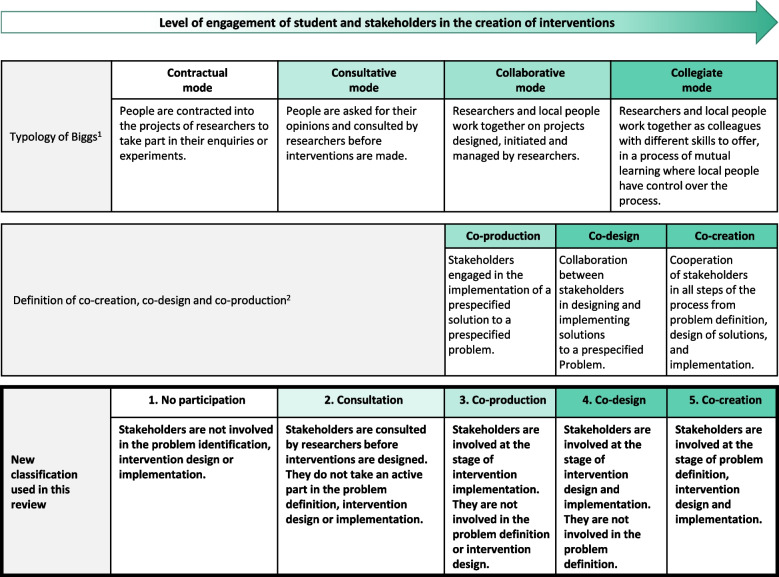
Classification of participatory and co-creation approaches Legend: ^1^ Typology of Biggs as presented in Cornwall and Jewkes (1995) [26]. ^2^ Co-creation, co-design and co-production as defined in Vargas (2022) [25]

We summarized participatory and co-creation approaches using two different but complementary frameworks presented in Fig. 1. First, we used the typology of Biggs [33], as reported in the article by Cornwall and Jewkes in 1995 [26], which has been used previously to analyze participatory approaches in nutrition research [31]. This typology describes four modes of participation of non-academic partners in the research process [26]. The modes of participation are considered as a continuum, where control over the research process gradually shifts from academic to non-academic partners. This classification has the advantage of proposing a consultative mode of participation in which non-academic partners are consulted, but not directly involved in the design or implementation of the intervention. Second, we used the definitions of co-production, co-design and co-creation as reported in the article by Vargas et al. in 2022 [25], where the engagement of non-academic partners in the problem identification and in the intervention design and implementation gradually increases across these three notions.

Then, based on these two previous frameworks, we proposed a five-level classification which we found more adapted to our specific research question and context. This classification describes a continuum of engagement from non-academic partners in the creation of interventions (Fig. 1). We defined partners as students and actors involved in the university management and the campus food environment. In level one called “No participation”, partners are not involved in any step of the intervention design or implementation. In level two called “Consultation”, partners are asked for their opinion by researchers before interventions are designed but do not take an active part in the definition of interventions. In level three called “Co-production”, partners are involved at the stage of intervention implementation, therefore at the late stage of the intervention process. In level four called “Co-design”, partners are also involved in the intervention design stage, meaning that they participate in designing and implementing a solution to a problem previously defined by the researchers. Finally, in level five called “Co-creation”, the most active form of collaboration, partners participate in the whole process, from the definition of the problem to intervention design and implementation.

### Quality assessment

We assessed study quality with the Effective Public Health Practice Project Quality Assessment Tool for Quantitative Studies 2003 [34]. This tool was developed to evaluate both randomized and non-randomized studies and provides a global rating of study quality using six components which can be rated as ‘strong’, ‘moderate’ or ‘weak’: A) selection bias, B) study design, C) confounders, D) blinding, E) data collection methods, and F) withdrawals and drop-outs. Because blinding of participants as well as evaluators is inherently difficult or impossible to achieve in nutritional intervention studies, we did not consider this dimension in the global scoring. The global quality score was defined as strong, moderate or weak if no component, one, or two or more components were rated as weak, respectively [34]. Quality of each included study was assessed independently by two reviewers (TA and AB). Any disagreement between the reviewers was resolved through discussion.

### Synthesis of effectiveness

In the included studies, effectiveness of interventions was reported either at the individual level (longitudinal evaluation, i.e. among the same participants before and after the intervention) or at the group level (i.e., repeated cross-sectional evaluations). When the study included a control group or a control location, we reported between-group comparisons only. Otherwise in interrupted time series design, we reported pre-post intra-group comparisons. Our analysis focused on short-term effectiveness. Studies with multiple post-intervention data collection were rare. Thus, we used the earliest post-intervention assessment for synthesis of effectiveness.

For each intervention arm, we reported the effectiveness of interventions on 5 categories of outcomes: self-reported overall diet quality score, consumption of food groups considered as healthy or unhealthy (e.g. fruit/vegetables and sugar-sweetened beverages, respectively) and food security assessed at the individual level; objectively-measured or self-reported food choices in campus food outlets assessed at the group level. For studies assessing two or more different parameters in a given category of outcomes (e.g. fruits and vegetables as well as whole grains), we considered the overall effect on this category as: 1) positive if a statistically significant improvement was found for at least one outcome, and no significant effect was found for other outcomes, 2) negative if a statistically significant deterioration was found for at least one outcome, and no significant effect was found for other outcomes, 3) null (no effect) if no significant change in any direction was found, 4) mixed if both a significant improvement and deterioration were found for different outcomes. Results were considered statistically significant when *p*-values reported by authors of included studies were < 0.05.

To synthetize effectiveness, we compared effectiveness of studies without participation (level 1) or with participation (levels 2–5). No meta-analysis could be conducted due to heterogeneity of study designs, intervention strategies and outcomes.

## Results

**Fig. 2:**
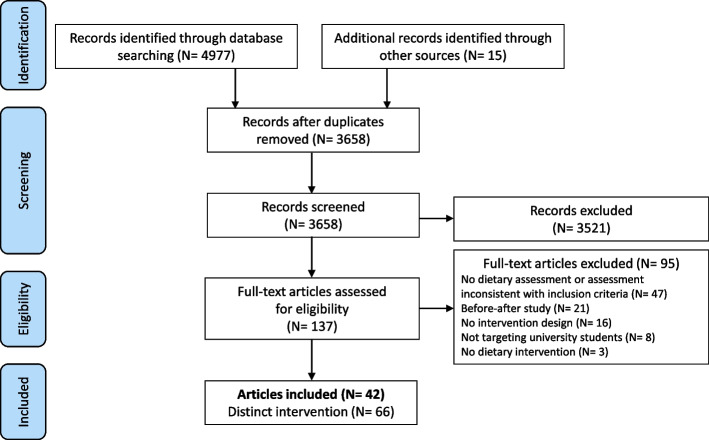
Systematic review flow diagram

The database search yielded 3658 articles after duplicates were removed (Fig. 2). The full text was retrieved from 137 articles, and 42 satisfied the inclusion criteria. Some articles compared in the same paper two or more interventions carried out at the same time in different groups or locations, and some articles implemented two or more consecutive interventions. Overall, 66 distinct interventions were described and included in the synthesis of findings.

### Study characteristics

Studies were conducted in 15 different countries. Most studies were conducted in North America (*N* = 23 in the USA and *N* = 2 in Canada). Seven studies were conducted in Europe (UK, Belgium, Spain, The Netherlands), 5 in Asia (China, Japan, India, Malaysia), 3 in South and Latin America (Peru, Puerto Rico, Chile), one in Oceania (Australia) and one in the Middle East (Turkey). The controlled design was used at the individual level in 26 studies: 21 randomized controlled trials (RCTs), five non-randomized controlled trials (non-RCTs). In seven studies, the controlled design was used at the group or building level, with (*N* = 5) or without (*N* = 2) randomization. The remaining nine studies used an interrupted time series design (i.e. two or more intervention phases interrupted by wash-out periods). The number of participants ranged from 23 [35] to 4208 [36] students and, for studies assessing food choices through sales data or observation, the number of meals or sales analyzed ranged from 260 [37] to 434,625 [38].

### Description of interventions

The main characteristics of interventions are summarized (articles by first author in alphabetical order) in Tables 1, 2, 3 and 4 and are described in detail in Supplementary Table 2. Overall, diet-only interventions were conducted in 28 (67%) of studies and combined interventions (i.e. targeting dietary intakes and other health behaviors) were conducted in 14 (33%) of studies. Diet interventions were classified as follows: education programs targeting individual dietary behavior conducted in 30 (45%) interventions, campus food environment interventions conducted in 29 (44%) interventions, food assistance programs aimed at improving food security conducted in five (8%) interventions, and multi-level interventions conducted in two (3%) interventions. Intervention duration was very short (< one month) for 23 (35%) interventions, short (one to < three months) for 20 (30%) interventions, intermediate (three to < six months) for eight (12%) interventions and long (> six months) for nine (14%) interventions. Intervention duration ranged from one unique session (one day) [35] to 10 months [39]. Duration was not reported for six (9%) interventions. Details regarding the intervention implementation such as who delivered the intervention, how and where, as well as strategies used to maintain fidelity, were reported in only very few studies (see Supplementary Table 2 for TIDierR checklist).

**Table 1:** Characteristics of education interventions in higher education settings (*N* = 26 studies and 30 intervention arms)

First author, date (reference)	Type of intervention					Topic	Duration of intervention	Participatory approach	
	**In-person education program**	**Digital education program**	**Cooking classes**	**Peer support**	**Financial incentives**			**Classification**	**Non-academic actors involved**
Bejar 2022 [40]		x				Diet	4 weeks	No participation	–
Blow 2022 [41]	x					Combined	2 weeks	No participation	–
Brown 2014 [42]		x				Diet	7 weeks	No participation	–
Brown 2014 [43]	x					Combined	20 weeks	No participation	–
Cameron 2015 [36]		x				Combined	NR	No participation	–
Dost 2022 [44]	x	x				Combined	6 months	No participation	–
Epton 2014 [45]		x				Combined	6 months	No participation	–
Halperin 2019 [46]	x			x		Combined	6 months	No participation	–
Hardan-Khalil 2022 [47]		x				Combined	8 weeks	No participation	–
Hayes 2020—Intervention 1 [48]		x				Diet	4 weeks	No participation	–
Hayes 2020—Intervention 2 [48]		x					4 weeks		
Hernández-Jaña 2020 [35]	x					Combined	1 session	No participation	–
Kattelmann 2014 [49]		x				Combined	10 weeks	Co-creation	Students, faculty staff
Krzyzanowski 2020 [50]		x				Combined	12 weeks	No participation	–
Lhakhang 2014—Intervention 1 [51]	x					Diet	NR	No participation	–
Lhakhang 2014—Intervention 2 [51]	x						NR		
Meng 2017 [52]		x				Diet	4 weeks	No participation	–
O'Brien 2016—Intervention 1 [53]		x				Diet	4 weeks	No participation	–
O'Brien 2016—Intervention 2 [53]		x					4 weeks		
Ohtsuki 2018 [54]	x		x			Diet	6 months	No participation	–
Patel 2020 [55]	x					Diet	2 weeks	No participation	–
Pope 2021—Intervention 2 [56]	x		x			Diet	6 weeks	No participation	–
Quintiliani 2016 [57]		x		x		Combined	8 weeks	Co-production	Students
Sandrick 2017 [58]	x	x				Combined	8 weeks	Consultation	Students
Schroeter 2021—Intervention 1 [59]	x					Diet	4 weeks	Co-production	Students
Schroeter 2021—Intervention 2 [59]	x				x		4 weeks		
Schweitzer 2016 [60]		x				Combined	6 months	No participation	–
Shahril 2013 [61]	x	x				Diet	10 weeks	Consultation	Students
Wang 2021 [62]		x				Combined	3 weeks	No participation	–
Whatnall 2019 [63]		x				Diet	NR	Co-creation	Students, faculty staff

Mixed interventions aimed at improving dietary intakes and other lifestyle habits (e.g., physical activity, stress, smoking…). When a study compared two interventions implemented at the same time in two different groups, interventions were named “interventions 1” and “interventions 2”. Abbreviations: NR, not reported

**Table 2:** Characteristics of campus food environment interventions in higher education settings (*N* = 13 studies and 29 intervention arms)

First author, date (reference)	Type of intervention					Topic	Duration of intervention	Participatory approach	
	**Food labeling**	**Food promotion**	**Food prices**	**Food provision**	**Food retail**			**Classification**	**Non-academic actors involved**
Cardenas 2015—Intervention phase 1 [37]		x			x	Diet	3 weeks	No participation	–
Cardenas 2015—Intervention phase 2 [37]		x	x		x		3 weeks		
Deliens 2016—Intervention phase 1 [64]		x	x			Diet	2 weeks	Co-production	Students
Deliens 2016—Intervention phase 2 [64]		x	x				2 weeks		
Dingman 2015 [65]		x				Diet	8 weeks	No participation	–
Lambert 2023—Intervention 1 [66]			x			Diet	14 weeks	No participation	–
Lambert 2023—Intervention 2 [66]	x		x				16 weeks		
Mistura 2019—Intervention phase 1 [67]		x		x		Diet	2 weeks	Consultation	Students, food services staff
Mistura 2019—Intervention phase 2 [67]		x		x			2 weeks		
Policastro 2017 [68]		x				Diet	4 weeks	No participation	–
Policastro 2017—Intervention phase 1 [69]		x				Diet	1 week	Consultation	Students
Policastro 2017—Intervention phase 2 [69]		x					1 week		
Policastro 2017—Intervention phase 3 [69]		x					1 week		
Schindler-Ruwisch 2021—Intervention phase 1 [70]		x				Diet	1 week	No participation	–
Schindler-Ruwisch 2021—Intervention phase 2 [70]		x		x			1 week		
Schindler-Ruwisch 2021—Intervention phase 3 [70]		x					1 week		
Seward 2016—Intervention 1 [38]				x	x	Diet	7 weeks	No participation	–
Seward 2016—Intervention 2 [38]	x	x		x	x		7 weeks		
Turnwald 2019—Intervention 1 [71]		x				Diet	NR	No participation	–
Turnwald 2019—Intervention 2 [71]		x					NR		
Van den Bogerd 2020—Intervention 1 [72]		x		x		Diet	3 weeks	Co-design	Students
Van den Bogerd 2020—Intervention 2 [72]		x		x			3 weeks		
Van den Bogerd 2020—Intervention 3 [72]		x		x			3 weeks		
Vermote 2020—Intervention phase 1 [73]		x				Diet	1 week	No participation	–
Vermote 2020—Intervention phase 2 [73]		x					1 week		
Vermote 2020—Intervention phase 3 [73]		x					1 week		
Vermote 2020—Intervention phase 4 [73]		x					1 week		
Walmsley 2018—Intervention phase 1 [39]					x	Diet	10 months	No participation	–
Walmsley 2018—Intervention phase 2 [39]					x		10 months		

When a study compared two interventions implemented at the same time in two different groups or locations, interventions were named “interventions 1” and “interventions 2”. When a study compared two consecutive interventions within the same group or location, interventions were named “intervention phase 1” and “intervention phase2”

*Abbreviations*: *NR* Not reported

**Table 3:** Characteristics of food assistance programs in higher education settings (*N* = 4 studies and 5 intervention arms)

First author, date (reference)	Topic	Duration of intervention	Participatory approach	
			**Type of intervention**	**Non-academic actors involved**
Gamba 2021—Intervention 1 [74]	Diet	16 weeks	No participation	–
Gamba 2021—Intervention 2 [74]		16 weeks		
Hernandez 2021 [75]	Diet	8 months	No participation	–
Nazmi 2022 [76]	Diet	1 year	No participation	–
Pope 2021—Intervention 3 [56]	Diet	6 weeks	No participation	–

When a study compared two interventions implemented at the same time in two different groups or locations, interventions were named “interventions 1” and “interventions 2”

**Table 4:** Characteristics of combined interventions in higher education settings (*N* = 2 studies and 2 intervention arms)

First author, date (reference)	Education interventions		Food assistance programs	Food environment interventions			Topic	Duration of intervention	Participatory approach	
	**In-person education program**	**Cooking classes**	**Food assistance programs**	**Food labeling**	**Food promotion**	**Food prices**			**Classification**	**Non-academic actors involved**
Lambert 2023—Intervention 3 [66]	x			x		x	Diet	14 weeks	No participation	–
Pope 2021—Intervention 1 [56]	x	x	x				Diet	12 weeks	No participation	–

When a study compared two interventions implemented at the same time in two different groups or locations, interventions were named “interventions 1” and “interventions 2”

### Description of participatory and co-creation approaches

The participatory and co-creation approaches are summarized in Tables 1, 2, 3 and 4 and described in detail in Supplementary Table 3. Overall, 32 (76%) studies did not use participatory and co-creation approaches. Among the 10 (24%) studies using a participatory and co-creation approach, six (60%) studies (corresponding to seven distinct interventions) implemented an education program [22, 49, 57–59, 61] (Table 1) and four (40%) studies (corresponding to 10 distinct interventions) conducted a campus food environment intervention [64, 67, 69, 72] (Table 2). None of them conducted a food assistance program. Four (10%) studies used a consultative approach [58, 61, 67, 69]. All of them consulted students using surveys, focus groups or pre-tests, and one study also conducted focus groups with food services staff [67]. Three (7%) studies used a co-production approach [57, 59, 64]. Students participated in the implementation of the intervention by providing information to other students in the on-campus food restaurant [64] and by acting as peer-counsellors or educator during educational programs [57, 59]. One (2%) study used a co-design approach [72]. Students were involved in the intervention design by participating in a brainstorming session and were also responsible for implementing the intervention [72]. Finally, two (5%) studies used a co-creation approach [22, 49]. In both studies, a steering committee composed of students and university staff members developed the intervention using the Predisposing, Reinforcing and Enabling Constructs in Educational Diagnosis and Evaluation (PRECEDE)—Policy, Regulatory, and Organizational Constructs in Educational and Environmental Development (PROCEED) participatory research model.

### Quality assessment

Summary results of study quality are presented in Table 5 and detailed results are presented in Supplementary Table 4. Among studies using a participatory and co-creation approach, study quality was rated as moderate in seven (70%) studies and as weak in three (30%) studies. Among those without participatory and co-creation approach, study quality was rated as strong in four (13%) studies, as moderate in 16 (50%) studies and as weak in 12 (38%) studies.

**Table 5:** Summary of results on quality assessment

	**Selection bias N (%)**	**Study design N (%)**	**Confounders N (%)**	**Data collection methods N (%)**	**Withdrawals and drop-outs N (%)**	**Global rating N (%)**
Strong quality	3 (7%)	29 (66%)	29 (66%)	24 (55%)	15 (34%)	5 (12%)
Moderate quality	18 (41%)	15 (34%)	2 (5%)	6 (14%)	20 (45%)	23 (52%)
Weak quality	23 (52%)	0 (0%)	13 (30%)	14 (32%)	9 (20%)	16 (36%)

### Effectiveness of interventions

#### Effectiveness assessed at the individual level

**Fig. 3:**
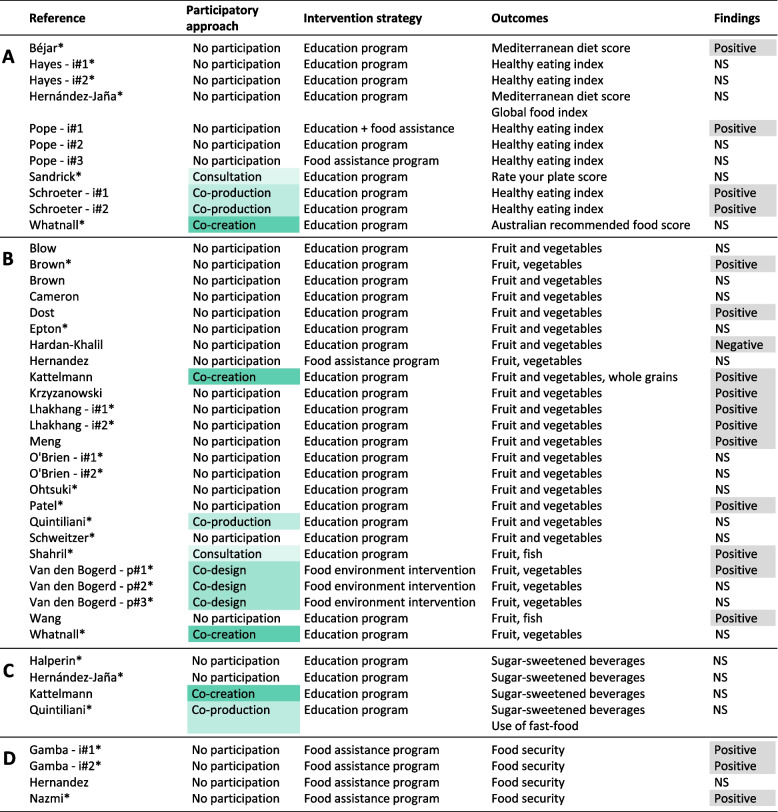
Synthesis of findings at the individual level on overall diet quality (**A**), healthy food group intake (**B**), unhealthy food group intake (**C**) and food security (**D**) Legend: * Study quality was rated as moderate or strong. i#1: intervention 1 (several interventions implemented at the same time in two different groups or locations). p#1: intervention phase 1 (several consecutive interventions within the same group or location)

A participatory and co-creation approach was used in 10/39 (26%) interventions assessing individual-level outcomes (Fig. 3 A-D). Among interventions using a participatory and co-creation approach, a positive effect of intervention was found on overall diet quality in 2/4 (50%) interventions, on the intake of healthy food groups in 3/7 (43%) interventions and on the intake of unhealthy food groups in 0/2 (0%) interventions. Among interventions without participation, a positive effect of intervention was found on overall diet quality in 2/7 (29%) interventions, on the intake of healthy food groups in 8/18 (44%) interventions and on the intake of unhealthy food groups in 0/2 (0%) interventions. None of the interventions assessing the effect of intervention on food security used a participatory and co-creation approach. Overall, independent of the type of individual-level outcome assessed, interventions using a participatory and co-creation approaches reported a positive effect in 5/13 (38%) cases (versus 13/31 or 42% for those without participation).

#### Effectiveness assessed at the group level

**Fig. 4:**
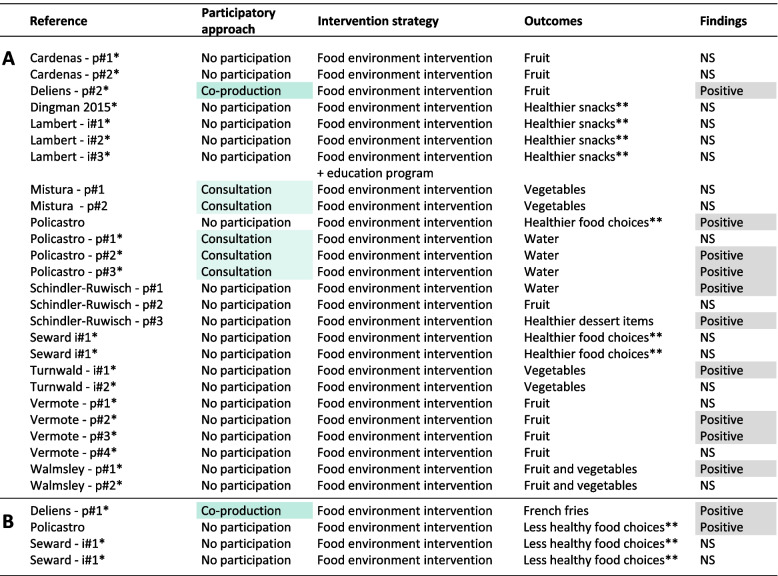
Synthesis of findings at the group level on healthier food choices (**A**) and less healthy food choices (**B**) Legend: * Study quality was rated as moderate or strong. ** Snacks or ingredients that were promoted during the intervention were considered as healthy food choices by authors of original studies. i#1: intervention 1 (several interventions implemented at the same time in two different groups or locations). p#1: intervention phase 1 (several consecutive interventions within the same group or location)

A participatory and co-creation approach was used in 7/26 (27%) interventions assessing group-level outcomes (Fig. 4 A-D). Among interventions using a participatory and co-creation approach, a positive effect of intervention was found on healthier food choices in campus food outlets in 3/6 (50%) interventions and on less healthy food choices in 1/1 (100%) interventions. Among interventions without participation, a positive effect of intervention was found on healthier food choices in 7/20 (35%) interventions and on less healthy food choices in 1/3 (33%) interventions. Overall, independent of the type of group-level outcome assessed, interventions using a participatory and co-creation approaches reported a positive effect in 4/7 (57%) cases (versus 8/23 or 35% for those without participation).

## Discussion

This systematic review examined 66 interventions, retrieved from 42 articles published since 2013, aimed at improving dietary intakes and/or food security in higher education students. Approximately a fourth of studies used a participatory and co-creation approach. These studies appeared to be more effective on students’ food choices in campus food outlets, with 57% of intervention arms reporting a positive finding (compared with 35% when no participatory and co-creation approach was used). The effect on individual dietary intakes and/or food security was however similar when comparing interventions with or without participatory and co-creation approaches (38% vs 42% of intervention arms with a positive finding, respectively). These results strengthen previous findings suggesting that a higher percentage of studies reported a positive effect on diet quality if they involved end-users in a participatory and co-creation approach, especially in the early stages of research design to identify the optimal intervention [31]. Our results should however be interpreted with caution given the limited number of studies using a participatory and co-creation approach, and the heterogeneity of studies in terms of study designs, interventions conducted and outcomes reported. Isolating the effect of participation and co-creation in this context is therefore challenging.

Studies using participatory and co-creation approaches carried out either education programs [22, 49, 57–59, 61] or campus food environment interventions [64, 67, 69, 72] but none of them carried out multi-level interventions targeting both the individuals and the food environment. The only intervention of this type identified in this review did not use participatory and co-creation approaches [66]. The rarity of multi-level nutritional interventions in the higher education setting contrasts with the variety of individual, interpersonal or environmental determinants of eating behavior that have been identified in this setting, such as lack of time, insufficient cooking skills, lack of financial resources, living away from the family home, or characteristics of the campus food environment [77, 78]. As in the general population [79], we can hypothesize that interventions targeting both the individual and environmental determinants of diet may be needed to further impact food habits of this population. This type of interventions however raises a number of operational and methodological challenges, including the need for teams made up of experts with diverse expertise from different organizations, the unpredictability of timelines or the lack of control over intervention implementation and changes in contextual variables [80, 81].

Participatory and co-creation approaches, which involve the beneficiaries and partners in the identification of problems and in the design and implementation of interventions, are thought to improve the relevance of interventions [23, 24]. Implementing multi-level nutrition interventions in the higher education setting would require the involvement of various partners, including students, university staff, on-campus food services staff, social organizations and, when appropriate, off-campus food retailers concerned with improving the healthiness of the broad campus food environment [30]. In this context, students are therefore considered as the direct beneficiaries of interventions, as well as potential partners of the participatory and co-creation process. Among the studies identified in this review, a majority of those using participatory and co-creation approaches involved only students in the co-creation process [57–59, 61, 64, 69], whereas only three studies also involved university and/or food services staff [49, 63, 67]. The nature of their involvement was very diverse, ranging from a simple consultation prior to the start of the intervention [58, 61, 67, 69] to an involvement from the earliest stages of problem definition and intervention design [49, 63]. We were therefore not able to infer how best to involve partners in this setting. Recent research suggests that trusting and respectful relationships, reciprocal acknowledgement between partners and flexibility were key practices in the co-creation process of health-enabling initiatives in food retail [82]. Involving partners in the entire research process, from problem identification through to intervention design, implementation and evaluation, has also been proposed to optimize the added-value of co-creation, although it is not a common practice in participatory nutrition interventions [83].

Demonstrating the added-value of participatory and co-creation approaches in the field of nutrition interventions is challenging [31]. Controlled trials providing formal comparisons of outcomes with and without participation and co-creation are indeed difficult to achieve [81, 84]. Co-created interventions are likely to be different in nature from other interventions, making it very difficult to isolate the impact of the participatory and co-creation protocol [84]. Besides, measuring the effectiveness of participation and co-creation raises in itself a number of challenges. Comparing solely predefined quantitative outcomes (e.g. dietary habits) before and after the intervention, as was done in the vast majority of studies included in this review, does not fit well with certain types of changes that can occur at any stage of the research process (e.g. partners engagement, improved relevance of research questions, co-creation of knowledge…) [85, 86]. Process evaluations, which can be performed through qualitative and quantitative methods (e.g. participant surveys, focus groups, meeting minutes, observations…) are valuable in addition to quantitative before-after evaluations [87]. The only two studies included in our review that used the most active form of co-creation also performed a formal process evaluation [49, 63]. In one study, students enrolled in the study who took part in the intervention were invited to reply to an online survey after the intervention to rate its quality and to report participation [49]. In the other, students were invited to reply to an online survey before the intervention to assess how they had been informed about the study [63]. Many other outcomes are interesting for a deeper understanding of the co-creation process, such as the diversity of participants, their engagement and their influence in decision-making, the number and types of events attended, the satisfaction with the process of participation, or the benefits and challenges of participation [87]. Obtaining these data is crucial for identifying best participatory and co-creation practices when designing, implementing and evaluating nutritional interventions in the higher education setting.

Although our review has several methodological strengths, some limitations should be mentioned. First, the heterogeneity in the study designs, the interventions conducted and the outcomes reported prevented us from performing a meta-analysis and to consider the effect size when analyzing the effectiveness of interventions. For similar reasons, we were not able to compare the effectiveness of interventions among those using a participatory and co-creation approach. Second, a number of studies under-reported important characteristics of interventions such as who delivered the intervention, how the intervention was tailored to the student population, as well as strategies used to maintain fidelity. Data extraction and interpretation of participatory and co-creation approaches were therefore limited. This limitation, often found in the field of lifestyle/non-pharmacologic interventions, makes the interpretation and replication of results difficult [32, 88]. Third, our literature search was limited to the peer-reviewed literature to ensure methodological rigor in the assessment of intervention effectiveness, and gray literature databases were not reviewed.

## Conclusions

Higher education campuses offer real-world experimental settings in which to implement and evaluate innovative nutrition interventions. Participation and co-creation approaches have been used in only a quarter of the 42 peer-reviewed studies included in this systematic review. Among them, 57% interventions reported a positive finding on food choices in campus food outlets (*vs* 35% in those not using participation and co-creation approaches); and 38% reported a positive finding on diet quality or food insecurity (*vs* 42% in those not using participation and co-creation approaches). Participation and co-creation approaches may therefore improve the effectiveness of such interventions but it has to be acknowledged the available evidence remains very limited with a small number of heterogeneous studies. This review points out there is a knowledge gap on how best to involve non-academic partners in the design and implementation of nutrition interventions in higher education settings. More research is therefore warranted to provide structured guidance on the practice of co-creation of nutrition interventions in this setting.

### Supplementary Information

Supplementary Material 1.

## Data Availability

All data generated or analyzed during this study are included in this published article and its supplementary information files.

## Abbreviations

Non-RCTs: Non-randomized controlled trials
PRECEDE: Predisposing, Reinforcing and Enabling Constructs in Educational Diagnosis and Evaluation
PRISMA: Preferred Reporting Items for Systematic Reviews and Meta-Analysis
PROCEED: Policy, Regulatory, and Organizational Constructs in Educational and Environmental Development RCTs: randomized controlled trials
TIDierR: Template for Intervention Description and Replication
