# Supplementary material for: Improving student diet and food security in higher education using participatory and co-creation approaches: a systematic review
Source: Int J Behav Nutr Phys Act. 2024 Jul 8;21:71. doi: 10.1186/s12966-024-01613-7 (PMC11232249; doi:10.1186/s12966-024-01613-7)
Supplement: Supplementary file 1 — Supplementary Material 1. [file 12966_2024_1613_MOESM1_ESM.docx]

**Supplementary Table 1 – Details of the keywords used to search articles in the different databases**

| **Database** | **Keywords** |
| --- | --- |
| PubMed | (university students[Title] OR college students[Title] OR university setting[Title] OR tertiary education OR college campus*[Title])  AND (diet*[Title/abstract] OR food[Title/abstract] OR eating[Title/abstract] OR nutrition*[Title/abstract] OR “food insecurity” [Title/abstract])  AND (intervention* OR program* OR participatory OR participation OR co-design OR codesign)  NOT (child*[Title] OR adolescent*[Title]) |
| Google scholar | “university students”\|“college students”\|“university setting”\|“tertiary education”\|“college campus diet\|food\|eating\|nutrition\|“food insecurity” intervention\|program\|participatory\|participation\|co-design\|codesign |
| Web of Science | ((((TI=(university students OR college students OR university setting OR tertiary education OR college campus*))) AND AB=(diet* OR food OR eating OR nutrition* OR “food insecurity”)) AND ALL=(intervention* OR program* OR participatory OR participation OR co-design OR codesign)) NOT TI=(child* OR adolescent*) |
| EMBASE | ('university students':ti,ab,kw OR 'college students':ti,ab,kw OR 'university setting':ti,ab,kw OR 'tertiary education':ti,ab,kw OR 'college campus*':ti,ab,kw) AND (diet*:ti,ab,kw OR food:ti,ab,kw OR eating:ti,ab,kw OR nutrition*:ti,ab,kw OR 'food insecurity':ti,ab,kw) AND (intervention* OR program* OR participatory OR 'participation'/exp OR participation OR 'co design' OR codesign) NOT (child*:ti OR adolescent*:ti) |

The term "food security" was not included in the list of key words because it includes the term "food", which has been included.

**Supplementary Table 2 – Main characteristics and TiDIER of the included intervention studies**

| First author  Country  Study design | Population | Intervention description | Who provided? How?  Where? | Tailoring and strategies used to maintain fidelity | Outcomes (measures)  Follow-up duration |
| --- | --- | --- | --- | --- | --- |
| Béjar 2022 (1)  Spain  Group-RCT  (class level) | Target population: students in Medicine and Pharmacy Participants:   - n= 283 (75% F):   158 in IG, 128 in CG | **Digital education program** Intervention group (28 days)   - Daily self-monitoring of food intake - Weekly feedback provided by a smartphone application on the 7 previous days, in the form of a score reflecting adherence to a Mediterranean diet - Two lists presenting 1) food groups consumed in sufficient quantity and 2) food groups consumed in insufficient quantities   Control group (28 days)   - Daily self-monitoring of dietary intakes | Who: NR  How: Smartphone application  Where: NA | Tailoring: NR  Strategies to maintain fidelity: study participation incentivized with the possibility of winning school materials (valued at EUR 500) in a raffle among participants who completed the study  Alert set up on telephones to inform participants that it was time to complete the app (participants allowed to set the time for the alert according to their own preferences) | Individual-level evaluation:   - Dietary intake (daily 19-item FFQ)   Follow-up duration:   - Post-intervention |
| Blow 2022 (2)  USA  RCT | Target population: students from psychology courses Participants:   - n= 235 (68% F) - Mean (SD) age: 20.7 (4.4) y - First-year students: 55% | **In-person education program** Intervention group (2 weeks)   - Addressing motivations and barriers to healthy food and physical activity - Integrating goal setting - Providing tips for healthy eating and physical activity   Control group (2 weeks)   - Self-monitoring of diet and physical activity during using paper food and activity logs | Who: NR  How: NR  Where: NR | Tailoring: the intervention focused on culturally-relevant food items that the participant enjoys  Strategies to maintain fidelity: participants were awarded 5h of course credit | Individual-level evaluation:   - Dietary intake (paper food log)   Follow-up duration:   - Post-intervention |
| Brown 2014 (3)  USA  RCT | Target population: 4000 undergraduate students Participants:   - n= 116 (gender NR) - Living off-campus: 66% | **Digital education program** Intervention group (7 weeks)   - 2 messages received each week consisting of the MyPlate icon along with 1 of the 7 behavior-directed motivational Dietary Guideline messages - Messages renewed every week   Control group (duration: NR)   - Brochure delivered at the beginning of the intervention containing the MyPlate icon along with the same 7 behavior-directed motivational Dietary Guideline messages | Who: messages developed with the US Department of Agriculture and Department of Health and Human Services  How: text messaging  Where: NA | Tailoring: NR  Strategies to maintain fidelity: participants in intervention and control groups received monetary compensation | Individual-level evaluation:   - Dietary intake (Rapid Eating FFQ)   Follow-up duration:   - Post-intervention |
| Brown 2014 (4)  Canada  Non-RCT | Target population: 380 students living within selected residence floors Population:   - n= 174 (58% F):   65 in IG, 109 in CG   - Mean (SD) age: 18.0 (1.0) y | **In-person education program** Intervention group (20 weeks)   - Structured activities provided periodically throughout the academic year, some incorporating behavior change techniques - Each HAL floor was supervised by a trained residence advisor who organized and facilitated activities and seminars related to behavioral nutrition, physical activity, and fostering social integration of students. They planned healthy active living experience (e.g. helped students organize intramural sport teams, initiated group activities and coordinated group challenges to prepare healthy meals) - One 1-hour small-group interactive workshop during the first semester with a physical activity counselor   Control group (20 weeks)   - Participated in standard residence community activities | Who: the counselor had received undergraduate and graduate training in physical activity behavior change  How: community interventions  Where: Student residences | Tailoring: the HAL community intervention was developed specifically to provide students with systematic exposure to in formation and develop knowledge related to healthy active lifestyle behaviors upon entry into university  Strategies to maintain fidelity: NR | Individual-level evaluation:   - F&V intake (2-item dietary questionnaire)   Follow-up duration:   - Post-intervention |
| Cameron 2015 (5)  UK  RCT | Target population: 5453 incoming undergraduate students not involved in a nutrition, exercise or kinesiology course Participants:   - n= 2 013 (72% F):   1 342 in IG, 1 272 in CG  Intervention group:   - Mean (SD) age: 18.7 (2.0) y   Control group:   - Mean (SD) age: 18.9 (2.7) y | **Digital education program** Intervention (duration NR)   - Self-affirmation manipulation task (students were asked to select their most important value, or provide their own, and to briefly explain why the value was important to them) - Online resources including theory-based messages (i.e., text, videos and links to further information) on diet, physical activity, smoking and alcohol intake and a planner containing instructions to form implementation intentions | Who: NR  How: NR  Where: NA | Tailoring: messages were developed on the basis of formative work that identified the key behavioral,  normative and control beliefs associated with intentions to perform each of the four health behaviors in new university students  Strategies to maintain fidelity: participants were entered into a £100 prize draw as an incentive for completing each questionnaire and  participants completing all three questionnaires received a £10 gift voucher and were entered into a further prize draw for an iPad Mini | Individual-level evaluation:   - F&V intake (2-item dietary questionnaire)   Follow-up duration:   - Intermediate term |
| Cardenas 2015 (6)  Peru  Interrupted time series | Target population: students visiting a university cafeteria Data:   - **260** meals sold during the study period | **Point-of-purchase strategic food positioning, informative/ motivational prompts and pricing intervention**  Intervention phase 1 -food positioning + informative/motivational prompts (3 weeks)   - Fruits repositioned into a covered container clearly displayed next to the point of purchase - Small sign stating "*Consuming five fruits and vegetables per day prevents many illnesses – World Health Organization*" and indicating the price per item of fruit placed on top of the container - Two posters (A4 size) with the same message and price placed at the entrance of the cafeteria and on the wall next to the point of purchase   Intervention phase 2 - food positioning + informative/educational prompts + pricing (3 weeks)   - Maintenance of intervention 1 + 33% price reduction on fruits | Who: NR  How: NA  Where: 1 university cafeteria | Tailoring: NR  Strategies to maintain fidelity: NA | Group-level evaluation:   - Food choices (sales data from a university cafeteria)   Follow-up duration:   - During intervention |
| Deliens 2016 (7)  Belgium  Interrupted time series | Target population: university students visiting an on-campus restaurant Data:   - n= 18364 students visiting the restaurant | **Pricing intervention and informative/motivational prompts** Intervention phase 1: pricing intervention on French fries (2 weeks)   - Week 1: extra-price of 0.5 € (= 10% of total meal price) for students who chose a selection of menus with French fries - Week 2: price increase of 1 € (= 20% of total meal price) for students who chose a selection of menus with French fries - During the intervention: three Master students involved helped serving the French fries wearing a special ‘French fries’ experiment’ t-shirt while providing additional information about the experiment   Intervention phase 2: pricing intervention on fruits (2 months after phase 1) (2 weeks)   - Week 1: price reduction of 0.5 € (= 10% of total meal price) for students who chose fruit for dessert - Week 2: price reduction of 1 € (= 20% of total meal price) for students who chose fruit for dessert   During phases 1 and 2, messages mentioning that prices had changed with the aim to help students make healthier food choices:   - Posters and information boards placed at the entrance of the restaurant and at the cash registers - Two posters and one information board placed at the stand where the French fries and/or fruits were served - A message about the experiments posted on the online university intranet platform and on Facebook | Who: NR  How: NA  Where: on-campus restaurant | Tailoring: NR  Strategies to maintain fidelity: NR | Group-level evaluation:   - Food choices (sales data from on-campus restaurant)   Follow-up duration:   - During intervention |
| Dingman 2015 (8)  USA  Group-RCT (vending machine level) | Target population: 4128 university students from 31 residence halls Participants: NA | **Informative/motivational prompts in food outlets** Intervention (4 weeks)   - Poster board adjacent to each vending machine listing the Nutrition Facts Panel (as required on packaged food in the US) for each product in that vending machine - 5 products in the vending machine highlighted with a sticker "better choice", which met certain nutrition criteria - Email sent to all students from the intervention residence about the Better Choice criteria   Control   - No intervention | Who: accuracy of the Better Choice criteria and email message were checked for accuracy by university and nutritionists in the community  How: NA  Where: on-campus residence hall | Tailoring: better choice was defined based on the Institute of Medicine front-of-Package Nutrition Rating Systems  Strategies to maintain fidelity: NA | Group-level evaluation:   - Food choices (sales data from vending machines)   Follow-up duration:   - During intervention |
| Dost 2022 (9)  Turkey  RCT | Target population: students enrolled in a formal education program on healthy lifestyle behaviors Participants:   - n= 110 (85% F): 55 in IG, 55 in CG   Intervention group:   - Mean (SD) age: 19.3 (1.8) y - Living with family: 70.9%   Control group:   - Mean (SD) age: 19.0 (1.0) y - Living with family: 70.9% | **In-person and digital education program**  Intervention group (8 weeks)   - Web-based health education: 1-h web‐based session per week covering topics related to healthy diet and physical activity (during 8 weeks) - Individual dietary and physical activity counseling - Self-monitoring of goal achievement (log diary for food, connected pedometer for physical activity) - Text messages and videos through social media groups: daily educational and motivational reminders, including a video of a student with obesity sharing his experience on weight loss, dietary and physical activity measures   Control group   - Educational booklet - Connected pedometer provided | Who: NR  How: web-based education, text messages and counseling  Where: NR for in-person intervention, online | Tailoring: text messages and videos were prepared according to the WHO obesity and physical activity training modules, Turkey Dietary Guideline, Turkish Journal of Diabetes and Obesity, and obesity and physical activity education modules of the Turkish Healthy Nutrition and Active Life program  Strategies to maintain fidelity: NR | Individual-level evaluation:   - Nutritional intake and food group consumption (chart) - F&V consumption (1-item FFQ)   Follow-up duration:   - Intermediate term |
| Epton 2014 (10)  UK  RCT | Target population: 4611 undergraduate students Participants:   - n= 1445 (58% F):   736 in IG, 709 in CG  Intervention group:   - Mean (SD) age: 18.8 (2.0) y   Control group:   - Mean (SD) age: 19.0 (2.9) y | **Digital education program** Intervention (6 months)   - Self-affirmation manipulation task (students were asked to select their most important value, or provide their own, and to briefly explain why the value was important to them) - Online and App-based resources including theory-based messages (i.e., text, videos and links to further information) on diet, physical activity, smoking and alcohol intake and a planner containing instructions to form implementation intentions | Who: NR  How: Online  Where: NA | Tailoring: NR  Strategies to maintain fidelity: 10 pounds compensation for filling all 3 questionnaires and 100 pounds prize draw | Individual-level evaluation:   - F&V intake (2-item dietary questionnaire)   Follow-up duration:   - Intermediate term |
| Gamba 2021 (11)  USA  RCT | Target population: college students with marginal food security Participants:   - n= 30 (76% F) - Aged 18 - 24 y: 76% | **Food assistance program** Intervention 1 (4 months)   - Months 1-2: $40 per month in the form of a gift card to a grocery store of their choice - Months 3-4: $40 per month in the form of a restaurant delivery service gift certificate   Intervention 2 (4 months)   - Months 1-2: $40 per month in the form of a restaurant delivery service gift certificate - Months 3-4: $40 per month in the form of a gift card to a grocery store of their choice | Who: NR  How: NR  Where: NR | Tailoring: NR  Strategies to maintain fidelity: NR | Individual-level evaluation:   - Food security status (Agriculture’s 10-item adult Food Security Survey Module)   Follow-up duration:   - Short term |
| Halperin 2019 (12)  Puerto Rica  RCT | Target population: undergraduate college students Participants:   - n= 39 (71.8% F) - Aged 18 y: 44% | **In-person peer-support education program** Intervention group (10 weeks)   - Weekly peer support groups (75 min each: identification and promotion of dietary and physical activity changes, techniques to reduce stress and increase mindfulness) based on components of the Health Self-empowerment Model   Control group (10 weeks)   - Basic educational resource materials on diet and physical activity | Who: Intervention developed by the research team:  - health behaviorist (trained in anthropology and epidemiology)  - PhD expert in nutritional interventions  - experienced yoga and meditation instructor who received additional training from the nutritional intervention’s expert  Sessions led by the yoga  and meditation  instructor (and by a registered nutritionist for the first 4 sessions)  How: groups of 9 members  Where: in an activity room at the college | Tailoring: NR  Strategies to maintain fidelity: students received 20$ for each measurements visit and 5$ for each group session | Individual-level evaluation:   - Dietary intakes (Multi-Cultural FFQ)   Follow-up duration:   - Intermediate term |
| Hardan-Khalil 2022 (13)  USA  Non-RCT | Target population: university students Participants:   - n= 201 (71% F) - Aged between 18-22 y: 91% | **Digital education program** Intervention group (8 weeks)   - Daily motivational text messages about nutrition and physical activity - Brochure containing nutrition and physical activity information materials   Control group   - Brochure containing nutrition and physical activity information materials | Who: research assistant  How: text messages were adapted from the 2019-2020 Dietary Guidelines for Americans  Where: NA | Tailoring: NR  Strategies to maintain fidelity: NA | Individual-level evaluation:   - F&V intake (2015 Food & Health Survey)   Follow-up duration:   - Post-intervention |
| Hayes 2020 (14)  USA  RCT | Target population: college students with overweight or obesity Participants:   - n= 95 (73% F):   31 in IG1, 34 in IG2, 30 in CG  Intervention group 1:   - Mean (SD) age: 21.2 (2.0) y - Mean (SD) BMI: 31.5 (4.8) kg/m^2^ - Living with family: 29%   Intervention group 2:   - Mean (SD) age: 20.7 (1.9) y - Mean (SD) BMI: 30.2 (4.7) kg/m^2^ - Living with family: 12%   Control group:   - Mean (SD) age: 20.7 (2.1) y - Mean (SD) BMI: 29.8 (3.9) kg/m^2^ - Living with family: 7% | **Digital education program** Intervention 1 - Implementation intention group (4 weeks)   - Six goals assigned: avoiding high-fat foods, making low-calorie substitutions, limiting portion size, avoiding sugar-sweetened beverages and other caloric drinks, and eating five servings of fruits and vegetables a day and measuring body weight on a daily basis - Formation of an implementation intention for each of the six goals   Intervention 2 - Enhanced implementation intention group (4 weeks)   - Six goals assigned: avoiding high-fat foods, making low-calorie substitutions, limiting portion size, avoiding sugar-sweetened beverages and other caloric drinks, and eating five servings of fruits and vegetables a day and measuring body weight on a daily basis - Formation of an implementation intention for each of the six goals - Online weekly fluency training - Text message reminders sent on 4 days each week of the intervention, containing all six implementation intention’s goal reminders that were obtained by asking participants to write down their reasons for wanting to lose weight   Control group (4 weeks)   - Six goals assigned: avoiding high-fat foods, making low-calorie substitutions, limiting portion size, avoiding sugar-sweetened beverages and other caloric drinks, and eating five servings of fruits and vegetables a day and measuring body weight on a daily basis | Who: NR  How: NR  Where: NA | Tailoring: NR  Strategies to maintain fidelity:  40$ compensation for participation in the study and possibility to win one of three 100$ prize if they answered to more than 85% of surveys | Individual-level evaluation:   - Dietary intakes (Diet History Questionnaire II FFQ)   Follow-up duration:   - Post-intervention |
| Hernandez 2021 (15)  USA  RCT | Target population: college students Participants:   - n= 1000 (67% F) - Mean (SD) age: 29.7 (10.4) y | **Food assistance program** Intervention (8 months)   - Food distributions on-campus, 2 times/month, each containing 60 pounds of perishable food items (fruits, vegetables, meat) and non-perishable food (dry goods) - Food was set up similar to a farmer’s market experience, and students were allowed to select their own food items | Who: NR  How: NR  Where: on campus | Tailoring: NR  Strategies to maintain fidelity:  25$ gift card for completion of each survey | Individual-level evaluation:   - Food security (USDA 18-item Food Security Module) - Dietary intakes (Block rapid food screener)   Follow-up duration:   - Post-intervention |
| Hernández-Jaña 2020 (16)  Chile  RCT | Target population: university students with overweight or obesity Participants:   - n= 23 (52% F) - Mean (SD) age: 20.9 (2.5) | **In-person education program** Intervention group (1 session)   - One 20-min single educational talk with two parts: general information (about obesity health risks, the benefits of a healthy lifestyle, distribution of meals, and the detrimental effect of a critical period such as the national holidays) and six specific recommendations (decrease food intake, reduce alcohol and sugary beverage consumption, increase physical activity levels, do not add mayonnaise, ketchup or salt to foods, consume only one typical food per day, and increase the ingest of vegetables)   Control group (1 session)   - One 20-min single educational talk with general talk and a general set of recommendations | Who: session provided by a nutrition specialist; recommendations defined by a committee of specialists  How: NR  Where: NR | Tailoring: NR  Strategies to maintain fidelity: NR | Individual-level evaluation:   - Dietary intakes (12-item global food index) - Mediterranean diet adherence (16-item KIDMED survey)   Follow-up duration:   - Post-intervention |
| Kattelmann 2014 (17)  USA  RCT | Target population: college students not involved in nutrition, exercise or health promotion courses Participants:   - n= 1639 (67% F) - Mean (SD) age: 19.3 (1.1) y - Living on campus: 74% | **Digital education program**  Intervention (10 weeks)   - 21 mini-educational lessons addressing eating behavior, physical activity, stress management, and healthy weight management through a non-diet approach - 3 weekly nudges delivered by e-mail messages: short, entertaining messages with videos personalized with the participant's name and stage-tailored to pre-contemplators, contemplators/ preparers, or actor/maintainers for fruit and vegetable consumption, physical activity, and stress management - 1 weekly nudge encouraging students to view the new lessons - students required to visit the website weekly to set goals for 1 or all 3 of targeted behavior(s), where they could view a graph of their goal(s), progress toward a goal, and recommendations for each target behavior   Follow-up phase (up to the 15-month follow-up)   - 4 monthly nudges delivered by email | Who: steering committees composed of the target audience (college students) and key health and wellness personnel (e.g. health and nutrition faculty, dining services staff, health services staff, recreation services staff, facilities and services administrators)  How: internet courses and email  Where: NA | Tailoring: tailored using the PRECEDE procedure involving the evaluation of 3 components: social; epidemiological, behavioral and environmental; educational and ecological  Strategies to maintain fidelity: NR | Individual-level evaluation:   - F&V intake (short-form National Cancer Institute F&V Screener) - Fat intake (short-form National Cancer Institute Fat Screener) - Sweetened-beverage, whole grain intake (FFQ)   Follow-up duration:   - Long term |
| Krzyzanowski 2020 (18)  USA  Non-RCT | Target population: university students Participants:   - n= 109 (76% F): 55 in IG, 54 in CG - Aged 18 y: 70% | **Digital education program** Intervention group (3 months)   - Traditional health cardiovascular disease risk-reduction course - App with 4 functions: dietary and physical activity self-tracking, health information, feedback and support modules   Control group (3 months)   - Traditional health course without the app | Who: NR  How: online  Where: NA | Tailoring: NR  Strategies to maintain fidelity: NR | Individual-level evaluation:   - F&V consumption (App-based self-monitoring)   Follow-up duration:   - Short term |
| Lambert 2023 (19)  USA  Group-based non-RCT | Target population: university students living in residence halls Data:   - n= 248 (55% F) (hall 1) - n= 260 (52% F) (hall 2) - n= 267 (62% F) (hall 3A) - n= 342 (60% F) (hall 3B) - n= 236 (51% F) (control hall) | **Pricing strategy, food labeling and education program** Intervention hall 1 - Pricing strategy (14 weeks)   - 25% price reduction of healthier snacks   Intervention hall 2 - Pricing strategy + food labeling (16 weeks)   - 25% price reduction of healthier snacks - Food labeling identifying healthier snacks - Posters located next to vending machines explaining the labeling and providing the nutrition facts label   Intervention hall 3 - Pricing strategy + food labeling + educational intervention (14 weeks including 4 weeks of nutrition education activities)   - 25% price reduction of healthier snacks - Food labeling identifying healthier snacks - Posters located next to vending machines explaining the labeling and providing the nutrition facts label - Two 2-h sessions per week including Interactive nutrition education activities implemented (targeting reading food labels, reducing salt, sugar, and fat content in snack foods)   Control hall   - No sales strategies or snack changes | Who: graduate students enrolled in a Nutrition Education and Behavioral Theory course developed and delivered the educational activities  How: online  Where: central corridors of the halls and student break rooms | Tailoring: educational topics were guided by the nutrient standards for Smart Snacks  Strategies to maintain fidelity: NA | Group-level evaluation:   - Food choices (sales data from vending machines)   Follow-up duration:   - During intervention |
| Lhakhang 2014 (20)  India  RCT | Target population: university students Participants:   - n= 205 (52% F) - Mean (SD) age: 20.7 (1.6) y | **In-person education program** Intervention group 1 (17 weeks)   - Motivational condition (package containing nutritional recommendations, risks associated with obesity, benefits and costs of action or inaction, prompt to intention formation) - Followed by the self-regulatory condition (instructions on how to perform the behavior, prompt for barrier identification and strategies to overcome these barriers, goal setting, behavioral planning)   Intervention group 2 (17 weeks)   - Self-regulatory condition (similar to intervention group 1) - Followed by the motivational condition (similar to intervention group 1) | Who: NR  How: NR  Where: NR | Tailoring: NR  Strategies to maintain fidelity: NR | Individual-level evaluation:   - F&V intake (2-item FFQ)   Follow-up duration:   - Post-intervention |
| Meng 2017 (21)  USA  RCT | Target population: undergraduate students Participants:   - n= 73 (67%) - Mean (SD) age: 19.9 (1.7) y | **Digital education program** Intervention group with 4 different conditions (4 weeks)   - Group-based self-tracking: participants joined a 4-person group wherein every group member posted self-tracking of their fruit and vegetable consumption 3 times a week. In each group, personal and self-tracking data from 3 members were experimentally manipulated as follows: - Demographically similar condition: ages, gender and ethnicities were identical to the participant - Demographically diverse condition: each confederate was different from the participant on two of the three demographic factors - Incremental-change model condition: average fruit and vegetable consumption of the 3 members were 3, 4, 4.5, and 5 servings in the 4 weeks - Ideal-change model condition: average fruit and vegetable consumption was 5 servings throughout the 4 weeks   Control group (4 weeks)   - Self-tracking of fruit and vegetable intake 3 times a week | Who: NR  How: digital self-tracking  Where: NA | Tailoring: NR  Strategies to maintain fidelity: 30$ for completing the study | Individual-level evaluation:   - F&V consumption (35-item F&V intake)   Follow-up duration:   - Post-intervention |
| Mistura 2019 (22)  Canada  Interrupted time series | Target population: undergraduate students Data:   - n= 5 098 purchases (baseline 1) - n= 6 173 purchases (intervention 1) - n= 5 754 purchases (baseline 2) - n= 7 385 purchases (intervention 2) | **Implementation of healthier food in food outlets with informative/motivational prompts** Intervention phase 1 (2 weeks)   - Addition of fresh, raw vegetables to the existing cooked vegetable option - Addition of a small poster displayed at eye level, with a hedonistic message (highlighting the addition of the fresh vegetable option with a colorful character) and an educational message (highlighting the health benefit of vegetable consumption)   Intervention phase 2 (2 weeks)   - Similar as phase 1 | Who: research staff  How: NA  Where: cafeteria | Tailoring: nudges co-designed with students  Strategies to maintain fidelity: NR | Group-level evaluation:   - Food choices (observations in a university cafeteria)   Follow-up duration:   - Post-intervention |
| Nazmi 2022 (23) USA  Non-RCT | Target population: college students with low income applying to a Supplemental Nutrition Assistance Program Participants:   - n= 70 (50% F) - Mean (SD) age: 21.3 (2.7) y | **Food assistance program** Intervention (duration NR)   - Enrollment in the Supplemental Nutrition Assistance Program (SNAP, known as CalFresh in California) - Details NR | Who: NR  How: NR  Where: NR | Tailoring: NR  Strategies to maintain fidelity:  gift card of 10$, 15 $ and 20$ for each survey completed | Individual-level evaluation:   - Food security (USDA Food Security Survey Module 6-Item Short Form)   Follow-up duration:   - Post-intervention |
| O'Brien 2016 (24)  USA  RCT | Target population: university students enrolled in introductory psychology classes Participants:   - n= 154 (68% F) - Mean (SD) age: 19.2 (1.2) y | **Digital education program** Intervention 1: web-based only group (1 month)   - On-screen, open-response prompts: students were encouraged to elaborate on the qualities that they personally value, and to imagine a version of their selves in the future that has attained high standards for health and fitness - Personal feedback including: comparison of behaviors to CDC guidelines, personal appraisals of dietary behaviors, discrepancy between current and ideal dietary behaviors, impact of dietary behaviors on health, encouragement and information, suggestions for healthy eating habits, prompts to identify personal preferences for healthy food choices, opportunity to anticipate and troubleshoot obstacles to attaining dietary goals - Presentation of on-campus resources   Intervention 2: web-based + message group (1 month)   - On-screen, open-response prompts (same as intervention 1) - Personal feedback (same as intervention 1) - Presentation of on-campus resources - Text messages during 30 days on: planning and monitoring, anticipation of barriers and plans to overcome barriers, peer-modeling and social support, development of specific implementation intentions to attain daily fitness and nutrition goals, encouragement and affirmation, educational information on health benefits of fruit and vegetable consumption   Control group   - No intervention | Who: NR  How: online  Where: NA | Tailoring: text messages were sent at a standard time of 4:30 p.m. in order to optimize its impact on late afternoon food choices and next-day planning and messages that included college-aged peer models were tailored by participant sex  Strategies to maintain fidelity: received course research credit to participate in the study | Individual-level evaluation:   - F&V intake (3-item FFQ based on the CDC - YRBSS) - Healthy food choices (FFQ based on healthy food available at the university restaurant)   Follow-up duration:   - Post-intervention |
| Ohtsuki 2018 (25)  Japan  RCT | Target population: university students not enrolled in nutritional sciences Participants:   - n= 104 (76% F) - Mean (SD) age: 20.6 (1.1) y - Mean (SD) BMI: 21.2 (3.4) kg/m^2^ - Living alone: 37 % | **Cooking classes with nutritional education** Intervention (25 weeks)   - Lesson on the significance of vegetable intake (general information on the benefits of vegetable intake) - 3-h tour of an agricultural farm tour (participation in farm activities, vegetable tasting) - Cooking class (preservation methods of vegetables, simple recipes to cook vegetables) | Who: NR  How: in person  Where: on campus and in local farms | Tailoring: the lesson focused on the benefits of vegetable intake on skin color, which might have more impact on young adults  Strategies to maintain fidelity: NR | Individual-level evaluation:   - Vegetable intake (diet history questionnaire)   Follow-up duration:   - Post-intervention |
| Patel 2020 (26)  India  Group-RCT (department level) | Target population: undergraduate women students Participants:   - n= 150 (100% W) - Mean (SD) age: 18.5 (0.5) y | **In-person education program** Intervention (2 weeks)   - Two 40-min educational sessions comprising nutrition cards, pamphlets (health benefits, recommended daily intake of fruit and vegetables and serving sizes), models and PowerPoint presentations (portion size, recommended daily intake of fruit and vegetables, cost of one serving, myths and misconceptions, and strategies | Who: NR  How: NR  Where: NR | Tailoring: NR  Strategies to maintain fidelity: NR | Individual-level evaluation:   - F&V intake (method NR)   Follow-up duration:   - Post-intervention |
| Policastro 2017 (27)  USA  Interrupted time series | Target population: university students Data:   - n = 9765 sales | **Informative/motivational prompts in food outlets** Intervention (4 weeks)   - Students ordered their sandwich using a modified “health-salient” order form, which highlighted the healthier ingredients by listing them first within each ingredient category, printing them in bolded, slightly larger font, and designating them with a star symbol)   Control (4 weeks)   - Students ordered their sandwich using the classical order form | Who: NR  How: NA  Where: on campus | Tailoring: NR  Strategies to maintain fidelity: NA | Group-level evaluation:   - Food choices (sales data from a university dining facility)   Follow-up duration:   - During intervention |
| Policastro 2017 (28)  USA  Interrupted time series | Target population: college students Data:   - n= 2393 students - n= 6730 meals sold | **Informative/motivational prompts in food outlets** Intervention phase 1 (1 week)   - Charity posters displayed at two fixed points in service areas within the dining establishment (orders and payment). Posters indicated that when choosing fountain water instead of another fountain beverage, the savings in food cost would be donated to a local hunger-focused charity   Intervention phase 2 (1 week)   - Calorie posters were displayed, indicating the energy content of sugar sweetened beverages   Intervention phase 3 (1 week)   - Posters with the charity-plus-calorie message were displayed | Who: NR  How: NA  Where: on campus dining hall | Tailoring: NR  Strategies to maintain fidelity: NA | Group-level evaluation:   - Food choices (sales data from a university dining facility)   Follow-up duration:   - During intervention |
| Pope 2021 (29)  USA  RCT | Target population: undergraduate students living off-campus with access to a kitchen Participants:   - n= 53 (77% F): 18 in IG1, 16 in IG2, 9 in IG3, 10 in CG   Intervention 1:   - Mean (SD) age: 20.6 (1.3) y   Intervention 2:   - Mean (SD) age: 20.9 (1.2) y   Intervention 3:   - Mean (SD) age: 20.6 (0.5) y | **Cooking classes and food assistance program** Intervention 1: cooking intervention with nutritional education followed by meal kit intervention (12 weeks: 6 weeks of cooking classes intervention + 6 weeks of meal kit boxes)   - 1 weekly cooking class including a brief lecture, a laboratory session in which participants worked in teams to actively practice skills and cook a meal, and ended with time to taste the meal and conduct a sensory analysis - Eight months after the cooking classes, meal kit boxes were delivered directly to the homes of participants, containing all of the ingredients and instructions needed to prepare 3 meals designed to feed two people. Each week, participants were able to select meals that suited their dietary needs (vegetarian, vegan, gluten-free)   Intervention 2: cooking intervention alone (6 weeks)   - 1 weekly cooking class (similar as intervention 1)   Intervention 3: meal kit intervention (6 weeks)   - Meal kit boxes (similar as intervention 1)   Control group  No intervention | Who: cooking classes taught by a chef educator trained in the food agency pedagogy  How: NR  Where: on campus and at-home delivery | Tailoring: cooking classes patterned after Dr. Amy Trubek’s food agency pedagogy  Strategies to maintain fidelity: Amazon gift cards, and gift cards to local stores, and kitchen tools (culinary knives, cookbooks, cookware, baking equipment, etc.) were offered to complete surveys at each time point | Individual-level evaluation:   - Dietary intakes (three 24-h food recall)   Follow-up duration:   - Post-intervention |
| Quintiliani 2016 (30)  USA  RCT | Target population: non-traditional undergraduate students (older, part-time enrollment, and working) Participants:   - n= 60 (58% F) - Mean (SD) age: 32.2 (10.0) y | **Digital peer-support education program**  Intervention group (8 weeks)   - Report sent by postal mail presenting baseline levels of diet and physical activity, recommendations, brief tips and links to health-related websites by postal mail - 3 telephone motivational interviewing-based counseling sessions with a trained student peer counselor: the guide was designed to be highly structured (providing wording examples, sentences...) but flexible enough to allow the peer counselors to draw on their unique perspective as a college student   Control group   - Report received by postal mail (same as intervention group) | Who: the counseling calls were directed by a written guide prepared by the study authors and were provided by student’s peer counselors previously trained  How: online  Where: NA | Tailoring: the counseling calls followed principles of motivational  interviewing, Social Contextual Model, and information obtained during qualitative formative research  Strategies to maintain fidelity:  $5 compensation for completing the screening and $75 for completing the follow-up questionnaires, students peer counselors received $50 for completing the training | Individual-level evaluation:   - F&V intake (7-item Block Food Screener) - Sugary drink intake (7-item Beverage Questionnaire) - Fast food intake (1-item question)   Follow-up duration:   - Post-intervention |
| Sandrick 2017 (31)  USA  RCT | Target population: university students Participants:   - n= 60 (68% W):   30 in IG, 30 in CG   - Mean (SD) age: 19.0 (1.0) - First-year students: 43% - Living on campus: 80% | **In-person and digital education program**  Intervention group (8 weeks)   - Feedback on baseline survey results to allow comparison with normative standards - 1-hour in-person educational session with a health coach design to set one goal for each behavioral domain (diet, exercise, stress management, sleep) - Brief behavioral assessments completed once a week - 3 weekly text messages sent by the health coach and personalized based on the brief behavioral assessments (3 types of messages: motivational, action-oriented, informational)   Control group   - Feedback on baseline survey results (same as intervention) but no other intervention | Who: health coaches who received a certification by the American Council on Exercise Supervision of the text message development was provided by the study’s principal investigator  How: face-to-face meetings and online  Where: NR | Tailoring: based on focus group results, a mix of action-oriented, informational, and motivational messages that were succinct, and sent no more than three times per week, were provided  Strategies to maintain fidelity: NR | Individual-level evaluation:   - Dietary intake (27-item Rate Your Plate survey)   Follow-up duration:   - Post-intervention |
| Schindler-Ruwisch 2021 (32)  USA  Interrupted time series | Target population: university students Participants:   - n= 4208 (66% F): 2 034 during intervention weeks, 2173 during control weeks | **Implementation of healthier food options in food outlets and informative/motivational prompts** Intervention phase 1: intervention on beverage (1 week)   - Signs promoting water and healthy beverage consumption instead of soda or juice displayed prominently at all the dining hall drink stations   Intervention phase 2: intervention on snacks (1 week)   - Addition of a fruit basket in the snack section of the dining halls - Signs promoting healthier snack consumption   Intervention phase 3: intervention on desserts (1 week)   - Signs promoting healthier dessert consumption | Who: survey items were reviewed by a registered dietician and 10 health science students for their applicability and  appropriateness  How: NA  Where: on-campus cafeteria/dining hall | Tailoring: NR  Strategies to maintain fidelity: NA | Group-level evaluation:   - Food choices in the campus restaurant (interviews)   Follow-up duration:   - During intervention |
| Schroeter 2021 (33)  USA  RCT | Target population: undergraduate college students not involved in a nutrition course  Participants:   - n= 57: 39 in IG, 18 in CG   Intervention groups   - Mean (SD) age: 16.6 (1.2) y   Control groups   - Mean (SD) age: 20.3 (1.7) y | **In-person education program** Intervention 1: education (4 weeks)   - 1 weekly 1-h education session focusing on the five food groups outlined in the USDA’s MyPlate (fruits, vegetables, grains, dairy, proteins) and including 3-4 quiz questions every 10-15 min   Intervention 2: education + incentives (4 weeks)   - Financial incentive of $500 in case the group improved their average healthy eating index by 5%   Control group   - No intervention | Who: sessions delivered by a professor and students in Food Science, Agribusiness and Agricultural Communication  How: group meetings  Where: NR | Tailoring: the education program was crafted specifically to capture target participants’ attention and encourage interaction and were adapted to students' eating habits  Strategies to maintain fidelity:  $5 gift cards for given to students who correctly answer questions from the verbally administered quizzes during the sessions | Individual-level evaluation:   - Dietary intakes (dietary recalls, ASA24)   Follow-up duration:   - Post-intervention |
| Schweitzer 2016 (34)  USA  RCT | Target population: college students Participants:   - n= 148 (69% F): 99 in IG, 49 in CG - Mean (SE) age: 19.7 (0.1) y | **Digital education program** Intervention group (24 weeks)   - Weekly, tailored, and interactive diet and physical activity goals - Feedback from baseline assessment - Weekly messages based on social and cognitive principles (goal setting with an emphasis small, achievable, and cumulative goals, focus on individual choice, direct information and goal relevance for each learner, overcoming barriers, specific action-based advice, salience of cues, building on prior learning, repetition of core messages, repeated practice of new behaviors) - Access to a personal account on the study website, where educational information and feedback on progress were offered   Control group (24 weeks)   - Feedback from baseline assessment - Received weekly information related to non-diet, non-exercise health topics (distracted driving, sleep hygiene, smoking cessation) | Who: NR  How: online  Where: NA | Tailoring: the web-based behavior change program created and managed by Nutrition Quest was modified by the authors for use with college students modifications primarily eliminated or replaced work and family-oriented language  Strategies to maintain fidelity: participants were given a $25 gift card to a local department store for completing baseline, week 12, and week 24 study visits.  Students who completed all visits were entered into a raffle for a $250 gift certificate | Individual-level evaluation:   - Dietary intake (adapted Block FFQ)   Follow-up duration:   - Post-intervention |
| Seward 2016 (35)  USA  Group-based non-RCT | Target population: undergraduate college student Data:   - n= 434625 meals served during the study | **Point-of-purchase strategic food positioning, implementation of healthier food, food labeling and informative/motivational prompts in food outlets** Intervention cafeterias 1: minimal intervention (7 weeks)   - Improved accessibility of healthier food and beverage items - Addition of water pitchers dispensing cucumber- or fruit-infused water   Intervention cafeterias 2: full intervention (7 weeks)   - Improved accessibility of healthier food and beverage items (same as intervention 1) - Addition of water pitchers dispensing cucumber- or fruit-infused water (same as intervention 1) - Traffic-light labeling (green, yellow, red labels) - Posters defining green labels as “nutrient-rich choice,” yellow labels as “nutrient-neutral choice,” and red labels as “more nutrient rich choice in green circle or yellow circle) - Stickers attached to cafeteria trays that visually displayed recommended portions of food types and the relative size of each food type on a plate   Control cafeterias   - No intervention | Who: NR  How: NA  Where: on-campus cafeterias | Tailoring: the labels followed university regulations prohibiting the posting of numerical nutrition facts, and they did not include calories as a criterion and stickers were based on “Healthy Plate” materials developed at the Harvard T. H. Chan School of Public Health  Strategies to maintain fidelity: NA | Group-level evaluation:   - Food choices (sales data from on-campus cafeterias)   Follow-up duration:   - During intervention |
| Shahril 2013 (36)  Malaysia  Group-RCT  (class level) | Target population: university students involved in management studies Participants:   - n= 417 (80% F): 205 in IG, 212 in CG   Intervention group   - Mean (SD) age: 19.0 (1.2) y   Control group   - Mean (SD) age: 19.2 (1.1) y | **In-person and digital education program**  Intervention group (10 weeks)   - 1.5-h conventional nutrition lecture by a nutrition expert addressing 3 themes ("Always be healthy", "Eat moderately" and "Live the future") - 3 sets of brochures as take-home messages, containing key recommendations and how to achieve the recommendations for each key message through three different themes - 13 text messages sent during the study period (1 every 5 days)   Control group   - No intervention | Who: lecture provided by a nutrition expert  How: group lecture and text messages  Where: on-campus and online | Tailoring: text messages were based on the latest Malaysian Dietary Guidelines  Strategies to maintain fidelity: NR | Individual-level evaluation:   - Dietary intake (diet history of the last 7 days)   Follow-up duration:   - Post-intervention |
| Turnwald 2019 (37)  USA  Group-RCT  (day level) | Target population: college student  Data:   - n= 185 days of data - n= 137842 individual diner decisions | **Informative/motivational prompts in food outlets** Intervention 1 (duration NR)   - Taste-focused signs posted above the vegetables, designed to elevate diners’ expectations of a positive taste experience with vegetables   Intervention 2 (duration NR)   - Health-focused signs posted above the vegetables, constructed using words that communicated nutritional qualities and health benefits of vegetables   Control (duration NR)   - Basic non-descriptive signs posted above the vegetables | Who: NR  How: NA  Where: on-campus dining hall | Tailoring: NR  Strategies to maintain fidelity: NA | Group-level evaluation:   - Food choices (sales data (observations in dining halls)   Follow-up duration:   - During intervention |
| Van den Bogerd 2020 (38)  The Netherlands  Interrupted time series | Target population: university student in applied sciences Participants:  Included in intervention 1   - n= 124 - Mean (SD) age: 19.7 (1.9) y - First-year students: 52%   Included in intervention 2   - n= 92 - Mean (SD) age: 19.6 (1.9) y - First-year students: 51%   Included in intervention 3   - n= 237 - Mean (SD) age: 18.8 (1.6) y - First-year students: 100% | **Implementation of free healthier food options in food outlets and informative/motivational prompts** Intervention 1: simple and quick (3 weeks)   - Addition of a stand with free fruits and vegetables (no serving limits or suggested amounts) - Posters with images of smiley fruits and vegetables figures located throughout the building   Intervention 2: fresh and natural version (3 weeks, starting 1 week after the end of intervention 1)   - Same stand - Potted plants placed on and around the stand, small carpet of artificial grass placed in front of the stand along with stools, bird sounds   Intervention 3: simple and quick (3 weeks)   - Same as intervention 1 - Free postcards displaying fruits and vegetables combined with catchy phrases were also offered at the stand - Messages displayed on information screens - Promotion of the stand on social media | Who: two fourth-year students  How: NA  Where: on campus | Tailoring: based on brainstorm and interviews with students, it was concluded that students preferred F&V to be offered in the afternoon and ‘ready to consume’  Strategies to maintain fidelity: students received a piece of fruit as a reward for their participation in the survey Free coffee or tea at the university and a power bank were raffled among the students who participated in the questionnaire at the post-tests | Individual-level evaluation:   - F&V intake (FFQ)   Follow-up duration:   - Short term |
| Vermote 2020 (39)  Belgium  Interrupted time series | Target population: college student and staff (separate analyses for students) Data:   - n= 12776 students ordering dessert (40% W) | **Informative/motivational prompts in food outlets** Intervention phase 1: posters (1 week)   - Posters of the Food Triangle (food pyramid) placed at the entrance and inside the restaurant and at the fruit stands   Intervention phase 2: posters + green heart icons (1 week)   - Same posters - Signs in the form of two green heart icons with a smiling face presented above the fruit stands   Intervention phase 3: posters + green heart icons + substitution message  (1 week)   - Same posters - Same green heart icons - Signs with a substitution message implying that less healthy choices should be replaced by fruit   Intervention phase 4: posters + green heart icons + substitution message + fruit norm (1 week)   - Same posters - Same green heart icons - Signs substitution messages - Sign with showing a "fruit norm message", i.e., which percent of customers chose fruit the week before | Who: NR  How: NA  Where: on-campus restaurant | Tailoring: NR  Strategies to maintain fidelity: NA | Group-level evaluation:   - Food choices (sales data from a university restaurant)   Follow-up duration:   - During intervention |
| Walmsley 2018 (40)  UK  Interrupted time series | Target population: college student Data:   - n= 93000 sales on average/week - n= 5564 fruit and vegetable sales | **Point-of-purchase strategic food positioning** Intervention phase 1 (40 weeks)   - Fruit and vegetables moved from the back of the store to the aisle closest to the entrance and also an entrance-facing display - Changes were made at the same time as a store renovation including changes to the store decoration and branding   Intervention phase 2 (40 weeks)   - Entrance-facing display of fruit and vegetables replaced with a refrigerated cabinet containing beverages (juices, smoothies, and sweet drinks) | Who: NR  How: NA  Where: on-campus grocery store | Tailoring: NR  Strategies to maintain fidelity: NR | Group-level evaluation:   - Food choices (sales data from a grocery store)   Follow-up duration:   - During intervention |
| Wang 2021 (41)  China  Non-RCT | Target population: undergraduate students involved in a nutrition and healthcare course Participants:   - n= 110 (59% F): 87 in IG, 23 in CG | **Digital education program** Intervention group (8 weeks)   - Involved in a WeChat group, received the weekly offline course of Application of Nutrition and Health Care - From week 5: received dietary advice, exercise encouragement, healthy habits reminders... (3 weeks)   Control group (8 weeks)   - Involved in a WeChat group (same as intervention group) - No other intervention | Who: dietitians were responsible for giving advice and dietary  consultation, bringing up nutrition topics.  Health assistant were responsible for reminders, feedbacks, group. atmosphere and to check uploading missions  The sport coach released a daily 5-min exercise task and replied to consultants  How: NA  Where: NA | Tailoring: health education contents specially designed for college students  Strategies to maintain fidelity: NR | Individual-level evaluation:   - Dietary intake (method NR)   Follow-up duration:   - Post-intervention |
| Whatnall 2019 (42)  Australia  RCT | Target population: university students Participants:   - n= 124 (73% W) - Mean (SD) age: 22.4 (4) y - Living off-campus: 84% | **Digital education program** Intervention group (duration NR)   - Brief web-based intervention focused on diet quality and including: 1) a brief screening quiz providing personalized feedback on eating behaviors and barriers to healthy eating, 2) provision of information, tips, and strategies for each target behavior and two guided exercises to facilitate behavior change, 3) goal setting, 4) creating strategies, 5) The website also provides resources including links and downloads to other reputable sources for further information   Control group (duration NR)   - Brief web-based existing intervention focused on alcohol | Who: NR  How: online  Where: NA | Tailoring: the development of the intervention was based on the PRECEDE-PROCEED participatory research model and draws on social cognitive theory and the theory of planned behavior  Strategies to maintain fidelity: students received $AU10 gift vouchers after completing baseline measures and after completing follow-up measures | Individual-level evaluation:   - Dietary intake (Australian Eating Survey FFQ)   Follow-up duration:   - Post-intervention |

Abbreviations: CG, control group; F, females; FFQ, food frequency questionnaire; F&V, fruits and vegetables; IG, intervention group; NA, not applicable; NR, not reported; SD, standard deviation; TIDieR, Template for Intervention Description and Replication.

**Supplementary Table 3. Description of participatory and co-creation approaches**

| **Reference** | **Description of participation** |
| --- | --- |
| **Studies using a consultative approach** | |
| Mistura 2016 (22) | **Students were** **surveyed** to understand what affects their decision to purchase vegetables. **Focus groups** **were conducted with food services staff** to identify which nudges, among a list previously defined by researchers, were feasible for implementation. |
| Policastro 2017 (28) | Poster messages were **pretested among students** to assess whether they would be likely to follow the recommendations provided in each poster and how likely they thought other students would follow the recommendations. |
| Sandrick 2017 (31) | **Focus groups** **were conducted with students** to understand their preferences regarding the text message content that are the basis of the intervention. |
| Shahril 2013 (36) | Intervention content (lectures, brochures, text messaging) were **pretested among university students** for clarity and readability. |
| **Studies using a co-production approach** | |
| Deliens 2016 (7) | During the first intervention phase, **3 Master students** were involved in the implementation of the intervention. They helped serving French fries wearing a special ‘French fries experiment’ t-shirt while **providing additional information** about the experiment. |
| Quintiliani 2016 (30) | After receiving a specific training, **students** acted as **peer counselors** during counseling sessions. A guide was written to help peer counselors providing the intervention. The guide was highly structured but flexible enough to allow the peer counselors to draw on their unique perspective as a college student. |
| Schroeter 2021 (33) | **Students from majors such as Food Science, Agribusiness and Agricultural Communication** delivered each session via in-person group meetings. |
| **Studies using a co-design approach** | |
| Van den Bogerd 2020 (38) | Before Intervention 1 started, a **brainstorm** with a group of six **first-year students** and **interviews with seven other first-year students** about the appearance and placement of the F&V stand were conducted. The brainstorm and interviews were conducted by **two fourth-year students** who were also responsible for the **implementation** of the intervention. They were responsible for filling the stand with F&V and keeping the stand clean, as well as being responsible for its appearance. |
| **Studies using a co-co-creation approach** | |
| Kattelmann 2014 (17) | The intervention was developed using the **community-based participatory research** (CPBR) process of PRECEDE-PROCEED. A steering committee composed of **college students** and **key health and wellness personnel** (eg health and nutrition faculty, dining services staff, health services staff, recreation services staff, facilities and services administrators) were formed at each institution to assist in each PRECEDE phase for **intervention design and implementation:**   - PRECEDE phase 1: social assessment to gather and evaluate information needed to guide the intervention development (focus groups). - PRECEDE phase 2: epidemiological, behavioral and environmental assessment to allow the incorporate environmental aspects in the intervention. - PRECEDE phase 3: educational and ecological assessment to evaluate how students viewed the importance and changeability of food habits and environmental factors. - PRECEDE phase 4: administrative and policy assessment and intervention alignment: teams of content experts used previous phases to develop the project. |
| Whatnall 2019 (42) | The intervention was developed using the **community-based participatory research** (CPBR) process of PRECEDE-PROCEED.  A steering committee was formed, with its role being to guide **intervention development**, assist in **piloting the intervention**, and to have ongoing involvement in ensuring **translation into University Health Promotion**. The steering committee included **key staff members** (eg, from University Health Promotion, Student Residences, and Student Communications and Marketing) and a **diverse group of students** representing undergraduate and postgraduate, health and non-health degree background, international and domestic, and male and female.   - PRECEDE phase 1: social assessment to develop an understanding of the target population and to explore its demographics, social norms, and health problems (literature review and steering committee meetings). - PRECEDE phase 2: epidemiological, behavioral and environmental assessment to identify the priority eating behaviors and determinants to be targeted and addressed in the intervention. - PRECEDE phase 3: educational and ecological assessment to evaluate the effectiveness of a potentially applicable intervention approach, and the steering committee was consulted for input. - PRECEDE phase 4: administrative and policy assessment and intervention alignment to determine the program and intervention components, with respect to the determinants of behavior identified in the previous phases and with consideration to the organizational, policy, and administrative resources available. |

**Supplementary Table 4. Quality of the included intervention studies**

| **Studies** | **Components** | | | | | **Global rating** |
| --- | --- | --- | --- | --- | --- | --- |
|  | **Selection bias** | **Study design** | **Confounders** | **Data collection methods** | **Withdrawals**  **and drop-outs** |  |
| Béjar 2022 (1) | Moderate | Strong | Strong | Strong | Strong | Strong |
| Blow 2022 (2) | Weak | Strong | Strong | Weak | Strong | Weak |
| Brown 2014 (3) | Weak | Strong | Strong | Strong | Moderate | Moderate |
| Brown 2014 (4) | Weak | Moderate | Weak | Weak | Weak | Weak |
| Cameron 2015 (5) | Weak | Strong | Strong | Moderate | Weak | Weak |
| Cardenas 2015 (6) | Moderate | Moderate | Weak | Strong | Moderate | Moderate |
| Deliens 2016 (7) | Moderate | Moderate | Weak | Strong | Moderate | Moderate |
| Dingman 2015 (8) | Moderate | Strong | Strong | Strong | Moderate | Strong |
| Dost 2022 (9) | Weak | Strong | Strong | Weak | Strong | Weak |
| Epton 2014 (10) | Weak | Strong | Strong | Moderate | Moderate | Moderate |
| Gamba 2021 (11) | Weak | Strong | Strong | Strong | Strong | Moderate |
| Halperin 2019 (12) | Weak | Strong | Strong | Strong | Strong | Moderate |
| Hardan-Khalil 2022 (13) | Weak | Strong | Weak | Strong | Weak | Weak |
| Hayes 2020 (14) | Moderate | Strong | Strong | Strong | Strong | Strong |
| Hernandez 2021 (15) | Weak | Strong | Strong | Strong | Weak | Weak |
| Hernández-Jaña 2020 (16) | Weak | Strong | Strong | Strong | Moderate | Moderate |
| Kattelmann 2014 (17) | Weak | Strong | Strong | Strong | Weak | Weak |
| Krzyzanowski 2020 (18) | Weak | Moderate | Strong | Weak | Weak | Weak |
| Lambert 2023 (19) | Moderate | Moderate | Weak | Strong | Moderate | Moderate |
| Lhakhang 2014 (20) | Strong | Strong | Strong | Weak | Strong | Moderate |
| Meng 2017 (21) | Weak | Strong | Strong | Weak | Moderate | Weak |
| Mistura 2019 (22) | Moderate | Moderate | Weak | Weak | Moderate | Weak |
| Nazmi 2022 (23) | Moderate | Strong | Weak | Strong | Moderate | Moderate |
| O'Brien 2016 (24) | Weak | Strong | Strong | Strong | Strong | Moderate |
| Ohtsuki 2018 (25) | Weak | Strong | Strong | Strong | Strong | Moderate |
| Patel 2020 (26) | Moderate | Strong | Strong | Weak | Strong | Moderate |
| Policastro 2017 (27) | Moderate | Moderate | Weak | Weak | Moderate | Weak |
| Policastro 2017 (28) | Moderate | Moderate | Strong | Strong | Moderate | Strong |
| Pope 2021 (29) | Weak | Strong | Strong | Strong | Weak | Weak |
| Quintiliani 2016 (30) | Weak | Strong | Strong | Strong | Strong | Moderate |
| Sandrick 2017 (31) | Weak | Strong | Strong | Strong | Strong | Moderate |
| Schindler-Ruwisch 2021 (32) | Moderate | Moderate | Weak | Weak | Moderate | Weak |
| Schroeter 2021 (33) | Weak | Strong | Strong | Strong | Weak | Weak |
| Schweitzer 2016 (34) | Weak | Strong | Strong | Strong | Strong | Moderate |
| Seward 2016 (35) | Strong | Moderate | Weak | Moderate | Moderate | Moderate |
| Shahril 2013 (36) | Moderate | Strong | Moderate | Weak | Strong | Moderate |
| Turnwald 2019 (37) | Moderate | Strong | Strong | Weak | Moderate | Moderate |
| Van den Bogerd 2020 (38) | Moderate | Moderate | Strong | Strong | Weak | Moderate |
| Vermote 2020 (39) | Moderate | Moderate | Moderate | Moderate | Moderate | Strong |
| Walmsley 2018 (40) | Moderate | Moderate | Weak | Moderate | Moderate | Moderate |
| Wang 2021 (41) | Weak | Strong | Strong | Weak | Moderate | Weak |
| Whatnall 2019 (42) | Weak | Strong | Strong | Strong | Strong | Moderate |

**References**

1. Bejar L, Garcia-Perea M, Mesa-Rodriguez P. Evaluation of an Application for Mobile Telephones (e-12HR) to Increase Adherence to the Mediterranean Diet in University Students: A Controlled, Randomized and Multicentric Study. Nutrients 2022;14:4196.

2. Blow J, Iii R, Cooper T. A pilot study examining the impact of a brief health education intervention on food choices and exercise in a Latinx college student sample. Appetite 2022;173:105979.

3. Brown O, O’Connor L, Savaiano D. Mobile MyPlate: A Pilot Study Using Text Messaging to Provide Nutrition Education and Promote Better Dietary Choices in College Students. J Am Coll Health 2014;62:320–7.

4. Brown DMY, Bray SR, Beatty KR, Kwan MYW. Healthy active living: a residence community-based intervention to increase physical activity and healthy eating during the transition to first-year university. J Am Coll Health 2014;62:234–42.

5. Cameron D, Epton T, Norman P, Sheeran P, Harris PR, Webb TL, Julious SA, Brennan A, Thomas C, Petroczi A, et al. A theory-based online health behaviour intervention for new university students (U@Uni: LifeGuide): Results from a repeat randomized controlled trial. Trials 2015;16:555.

6. Cardenas MK, Benziger CP, Pillay TD, Miranda JJ. The effect of changes in visibility and price on fruit purchasing at a university cafeteria in Lima, Peru. Public Health Nutr 2015;18:2742–9.

7. Deliens T, Deforche B, Annemans L, De Bourdeaudhuij I, Clarys P. Effectiveness of Pricing Strategies on French Fries and Fruit Purchases among University Students: Results from an On-Campus Restaurant Experiment. PLoS One 2016;11:e0165298.

8. Dingman DA, Schulz MR, Wyrick DL, Bibeau DL, Gupta SN. Does providing nutrition information at vending machines reduce calories per item sold? J Public Health Policy 2015;36:110–22.

9. Dost A, Esin M. Effects of the UNI-PAHNP on physical activity and nutrition behaviors in overweight/obese university students in Turkey. Perspect Psychiatr Care 2022;58:2003–16.

10. Epton T, Norman P, Dadzie A, Harris P, Webb T, Sheeran P, Julious S, Ciravegna F, Brennan A, Meier P, et al. A theory-based online health behaviour intervention for new university students (U@Uni): results from a randomised controlled trial. BMC Public Health 2014;14.

11. Gamba R, Wood L, Ampil A, Engelman A, Lam J, Schmeltz M, Pritchard M, Santillan J, Rivera E, Ortiz N, et al. Investigating the Feasibility of a Restaurant Delivery Service to Improve Food Security among College Students Experiencing Marginal Food Security, a Head-to-Head Trial with Grocery Store Gift Cards. Int J Environ Res Public Health 2021;18:9680.

12. Halperin DT, Laux J, LeFranc-García C, Araujo C, Palacios C. Findings From a Randomized Trial of Weight Gain Prevention Among Overweight Puerto Rican Young Adults. J Nutr Educ Behav 2019;51:205–16.

13. Hardan-Khalil K, Costa C, Fisher D. Daily motivational text messages impact on college students’ nutritional awareness and physical activity levels. J Am Coll Health 2022;70:2511–8.

14. Hayes JF, Balantekin KN, Graham AK, Strube MJ, Bickel WK, Wilfley DE. Implementation intentions for weight loss in college students with overweight and obesity: a proof-of-concept randomized controlled trial. Transl Behav Med 2021;11:359–68.

15. Hernandez DC, Daundasekara SS, Walton QL, Eigege CY, Marshall AN. Feasibility of Delivering an on-Campus Food Distribution Program in a Community College Setting: A Mixed Methods Sequential Explanatory Investigation. Int J Environ Res Public Health 2021;18:12106.

16. Hernandez-Jana S, Huber-Perez T, Palma-Leal X, Guerrero-Ibacache P, Campos-Nunez V, Zavala-Crichton J, Jorquera-Aguilera C, Sadarangani K, Rodriguez-Rodriguez F, Cristi-Montero C. Effect of a Single Nutritional Intervention Previous to a Critical Period of Fat Gain in University Students with Overweight and Obesity: A Randomized Controlled Trial. Int J Environ Res Public Health 2020;17:5149.

17. Kattelmann KK, Bredbenner CB, White AA, Greene GW, Hoerr SL, Kidd T, Colby S, Horacek TM, Phillips BW, Koenings MM, et al. The effects of Young Adults Eating and Active for Health (YEAH): a theory-based Web-delivered intervention. J Nutr Educ Behav 2014;46:S27–41.

18. Krzyzanowski M, Kizakevich P, Duren-Winfield V, Eckhoff R, Hampton J, Carr L, McCauley G, Roberson K, Onsomu E, Williams J, et al. Rams Have Heart, a Mobile App Tracking Activity and Fruit and Vegetable Consumption to Support the Cardiovascular Health of College Students: Development and Usability Study. JMIR Mhealth Uhealth 2020;8:15156.

19. Lambert L, Mann G, Knight S, Partacz M, Jurss M, Eady M. Impact of Smart Snacks Intervention on College Students’ Vending Selections. J Am Coll Health 2023;71:952–8.

20. Lhakhang P, Godinho C, Knoll N, Schwarzer R. A brief intervention increases fruit and vegetable intake. A comparison of two intervention sequences. Appetite 2014;82:103–10.

21. Meng J, Peng W, Shin SY, Chung M. Online Self-Tracking Groups to Increase Fruit and Vegetable Intake: A Small-Scale Study on Mechanisms of Group Effect on Behavior Change. J Med Internet Res 2017;19:e63.

22. Mistura M, Fetterly N, Rhodes R, Tomlin D, Naylor P. Examining the Efficacy of a "Feasible’ Nudge Intervention to Increase the Purchase of Vegetables by First Year University Students (17-19 Years of Age) in British Columbia: A Pilot Study. Nutrients 2019;11:1786.

23. Nazmi A, Condron K, Tseng M, Volpe R, Rodriguez L, Lopez M, Martinez S, Freudenberg N, Bianco S. SNAP Participation Decreases Food Insecurity among California Public University Students: A quasi-experimental Study. Journal of Hunger & Environmental Nutrition 2022;18:123–38.

24. O’Brien LM, Palfai TP. Efficacy of a brief web-based intervention with and without SMS to enhance healthy eating behaviors among university students. Eat Behav 2016;23:104–9.

25. Ohtsuki M, Shibata K, Fukuwatari T, Sasaki Y, Nakai K. Randomized controlled trial of educational intervention to increase consumption of vegetables by Japanese university students. Health educ 2018;118:290–303.

26. Patel N, Lakshminarayanan S, Olickal JJ. Effectiveness of nutrition education in improving fruit and vegetable consumption among selected college students in urban Puducherry, South India. A pre-post intervention study. Int J Adolesc Med Health 2022;34:243–8.

27. Policastro P, Smith Z, Chapman G. Put the healthy item first: Order of ingredient listing influences consumer selection. J Health Psychol 2017;22:853–63.

28. Policastro P, Palm T, Schwartz J, Chapman G. Targeted Calorie Message Promotes Healthy Beverage Consumption Better than Charity Incentive. Obesity (Silver Spring) 2017;25:1428–34.

29. Pope L, Alpaugh M, Trubek A, Skelly J, Harvey J. Beyond Ramen: Investigating Methods to Improve Food Agency among College Students. Nutrients 2021;13:1674.

30. Quintiliani L, Whiteley J. Results of a Nutrition and Physical Activity Peer Counseling Intervention among Nontraditional College Students. J Cancer Educ 2016;31:366–74.

31. Sandrick J, Tracy D, Eliasson A, Roth A, Bartel J, Simko M, Bowman T, Harouse-Bell K, Kashani M, Vernalis M. Effect of a Counseling Session Bolstered by Text Messaging on Self-Selected Health Behaviors in College Students: A Preliminary Randomized Controlled Trial. JMIR Mhealth Uhealth 2017;5:e67.

32. Schindler-Ruwisch J, Gordon M. Nudging healthy college dining hall choices using behavioral economics. J Am Coll Health 2021;69:697–703.

33. Schroeter C, Corder T, Brookes B, Reller V. An incentive-based health program using MyPlate: a pilot study analyzing college students? dietary intake behavior. J Am Coll Health 2021;69:252–9.

34. Schweitzer A, Ross J, Klein C, Lei K, Mackey E. An Electronic Wellness Program to Improve Diet and Exercise in College Students: A Pilot Study. JMIR Res Protoc 2016;5:e29.

35. Seward MW, Block JP, Chatterjee A. A Traffic-Light Label Intervention and Dietary Choices in College Cafeterias. Am J Public Health 2016;106:1808–14.

36. Shahril MR, Wan Dali WPE, Lua PL. A 10-Week Multimodal Nutrition Education Intervention Improves Dietary Intake among University Students: Cluster Randomised Controlled Trial. J Nutr Metab 2013;2013:658642.

37. Turnwald BP, Bertoldo JD, Perry MA, Policastro P, Timmons M, Bosso C, Connors P, Valgenti RT, Pine L, Challamel G, et al. Increasing Vegetable Intake by Emphasizing Tasty and Enjoyable Attributes: A Randomized Controlled Multisite Intervention for Taste-Focused Labeling. Psychol Sci 2019;30:1603–15.

38. van den Bogerd N, Peppelenbos H, Leufkens R, Seidell JC, Maas J, Dijkstra SC. A free-produce stand on campus: impact on fruit and vegetable intake in Dutch university students. Public Health Nutr 2020;23:924–34.

39. Vermote M, Nys J, Versele V, D’Hondt E, Deforche B, Clarys P, Deliens T. The effect of nudges aligned with the renewed Flemish Food Triangle on the purchase of fresh fruits: An on-campus restaurant experiment. Appetite 2020;144:104479.

40. Walmsley R, Jenkinson D, Saunders I, Howard T, Oyebode O. Choice architecture modifies fruit and vegetable purchasing in a university campus grocery store: time series modelling of a natural experiment. BMC Public Health 2018;18:1149.

41. Wang M, Guo Y, Zhang Y, Xie S, Yu Z, Luo J, Zhang D, Ming Z, Li X, Yang M. Promoting healthy lifestyle in Chinese college students: evaluation of a social media-based intervention applying the RE-AIM framework. Eur J Clin Nutr 2021;75:335–44.

42. Whatnall M, Patterson A, Chiu S, Oldmeadow C, Hutchesson M. Feasibility and Preliminary Efficacy of the Eating Advice to Students (EATS) Brief Web-Based Nutrition Intervention for Young Adult University Students: A Pilot Randomized Controlled Trial. Nutrients 2019;11:905.
